# The role of an aromatic group in remote chiral induction during conjugate addition of α-sulfonylallylic carbanions to ethyl crotonate

**DOI:** 10.3762/bjoc.4.32

**Published:** 2008-09-23

**Authors:** Shlomo Levinger, Ranjeet Nair, Alfred Hassner

**Affiliations:** 1 Department of Chemistry, Bar-Ilan University, Ramat-Gan 52900, Israel

**Keywords:** cation–π interaction, conjugate addition, diastereoselectivity, regioselectivity, remote chiral induction

## Abstract

The impact of a remote aromatic nucleus on the stereochemical outcome of the conjugate addition of α-sulfonylallylic carbanions to an α,β-unsaturated ester was investigated. α-Regioselectivity coupled with *anti*-diastereoselectivity is accompanied by a prominent preference for relative configuration **3** over **4**. The 9-anthryl moiety has shown itself greatly superior over all other groups in this bias. A lithium ion–aromatic π interaction has been postulated as decisive for the remote transmission of chirality.

## Introduction

**Scheme 1:**
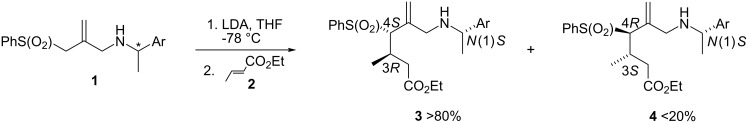
Transmission of asymmetry in the conjugate addition of allyl sulfones to ethyl crotonate depending on the presence of a remote aromatic nucleus.

Recently we have disclosed a pathway for remote asymmetric induction in conjugate additions involving lithiated α-(phenylsulfonyl)allylic carbanions bearing a chiral auxiliary [1]. Transmission of asymmetry, though four atoms removed, was striking and was found to depend on the presence of an aromatic nucleus bound to the stereogenic center attached to N in **1**. Thus, addition to crotonate **2** of the lithio derivative of amino-substituted allyl sulfone **1**, containing a stereogenic center, proceeded with diastereoselectivity of greater than 8:2 (ratio of **3** to **4**), when the Ar substituent in **1** was phenyl or 1-naphthyl (Scheme 1). On the other hand, when this substituent (Ar in Scheme 1) was cyclohexyl, no selectivity at all was observed (dr 1:1).

We had proposed that π–Li^+^ interaction may be responsible for this phenomenon. We had shown earlier [2] that conjugate addition of lithiated allyl sulfone **5** to unsaturated esters of type **2** takes place α-regioselectively and almost exclusively in an *anti* fashion (where the *syn* and *anti* designations are based on the extended form including both anion stabilizing groups [3]). Hence, it was not surprising that both diastereomeric products, **3** and **4**, of the addition of **1** to **2**, possessed the *anti* configuration. Starting with the *S* configuration in amine **1**, the major isomer was proved (in one case by X-ray analysis) to have the 3*R*,4*S* absolute configuration [1].

It was important to examine the effect of other aromatic substituents Ar in amino-substituted sulfone **1** and their influence on this remote induction of chirality, in the hope of further improving the remote diastereoselectivity. We report herein on the relationship between the nature of the aromatic nucleus and the extent of diastereoselectivity during the addition of **1** to ethyl *trans*-crotonate (**2**); we show that essentially 100% chiral induction can be achieved in the case of the 9-anthryl derivative.

## Results and Discussion

**Scheme 2:**
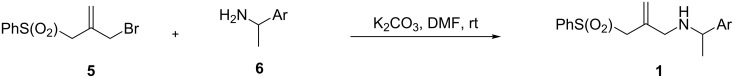
Preparation of donor precursors for conjugate addition (**1**), bearing a remote stereogenic center.

A series of α-sulfonylallylic donor precursors **1**, bearing a remote stereogenic center, were prepared by condensing a chiral aryl- or heteroarylalkylamine **6** with the bromo-substituted allyl sulfone **5** [2] (Scheme 2 and Table 1).

**Table 1:** Preparation of α-sulfonylallylic donor precursors **1** according to Scheme 2.

Product **1**	Ar	Yield (%)^a^	Mp (°C)

**a**	Phenyl^b^	86	58–59
**b**	4-Methoxyphenyl	92	60–61
**c**	4-Nitrophenyl	68	68–69
**d**	2-Thienyl	48	64–65
**e**	1-Naphthyl	100	oil
**f**	2-Naphthyl	63	73–74
**g**	2-Phenanthryl	63	oil
**h**	9-Anthryl	80	oil
**i**	2-Anthryl	59	143–145
**j**	Mesityl	40	oil

^a^Refers to the purified product, ^b^[4].

**Scheme 3:**
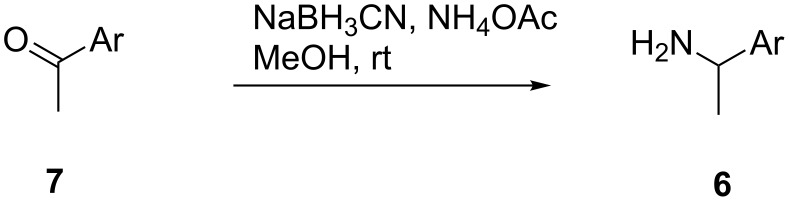
Borch reductive amination of acetophenones.

Of the primary amines **6** used for generation of amino-substituted sulfones **1**, 1-(2-thienyl)ethanamine (**6d**) was prepared from 2-acetylthiophene **7d** by a modified Leuckart reaction [5–6], while amines **6b**, **6f**, **6g** and **6i** were obtained through Borch reductive amination of the corresponding methyl ketones **7** with sodium cyanoborohydride and ammonium acetate in methanol (Scheme 3) [7–8].

9-Acetylanthracene does not lend itself to the Borch reductive amination, which entails a nucleophilic addition step of ammonia (or amine) to the carbonyl function [7]. Conjugation between the anthracene nucleus and an acyl substituent at position 9 is sterically inhibited by the flanking hydrogen atoms residing on centers 1 and 8, thus forcing the carbonyl plane into orthogonality with the aromatic ring, as has been demonstrated spectroscopically [9]. Such a conformation prevents sterically a potential nucleophile from efficiently approaching the carbonyl bond as is evidenced from the inertness of 9-acylanthracenes toward typical reagents attacking the carbonyl function like hydroxylamine and semicarbazide [9].

**Scheme 4:**
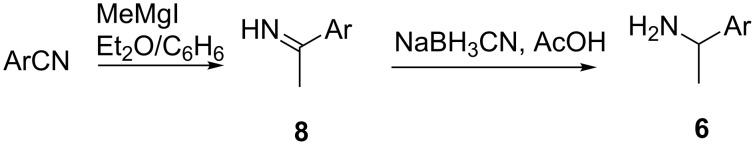
Preparation of [(9-anthryl)alkyl]- and (mesitylalkyl)amines **6h** and **6j** from nitriles via imines **8**.

Consequently, our approach toward both the (9-anthryl)- and the mesitylalkylamines **6h** and **6j** was based on the corresponding arenecarbonitriles, which were reacted upon by methylmagnesium iodide to give the isolable imines **8** [9–10] (Scheme 4). The stability of these imines toward hydrolysis may be accounted for by steric hindrance to the attack of a nucleophile on a trigonal carbon whose plane is perpendicular to the bis-*ortho*-aromatic ring to which it is attached. The preference for orthogonality between the planar function and the aromatic nucleus in the nitrogen derivatives of 9-acylanthracenes has been corroborated spectroscopically (lack of conjugation in the semicarbazide and the oxime). Furthermore, steric hindrance to attack at the cyano group of anthracene-9-carbonitrile was indicated by formation of a 10-*tert*-butyl derivative on reaction with *tert*-butylmagnesium chloride [9]. However, since iminium ion intermediates are much more reactive than ketones [7], imines **8h** and **8j** were successfully reduced to the primary amines **6** upon treatment with sodium cyanoborohydride in an acidic medium.

Phenyl-, (4-nitrophenyl)- and (1-naphthyl)ethylamines **6a**, **c**, **e** are commercial.

Interestingly, the 9-anthryl nucleus in both primary amine **6h** and its allyl sulfone congener **1h** shows a peculiar ^1^H NMR spectrum: a broad signal representing both protons 1 and 8 in the first compound **6h** and two distinct broad signals for the same protons in **1h** indicating an ongoing dynamic process. We believe this to be a manifestation of restricted rotation around the bond connecting the 9-anthryl group to the tetrahedral amino-substituted carbon atom due to steric hindrance exerted by protons 1 and 8 – similarly to the factors behind the special spectroscopic and chemical behaviour of 9-acylanthracenes and their derivatives, described above. The appearance of H-1 and H-8 resonances of 1-(9-anthryl)ethylamines **6h** and **1h** at a prominently low field (δ 9.2–8.4 compared to δ 7.91 for anthracene) is also worth noting in this context. Restricted rotation in acylnaphthalenes has been extensively studied [11].

Amino-substituted allyl sulfones **1** were deprotonated with LDA at −78 °C in THF and the lithio derivative obtained was allowed to react for a specified time at that temperature with ethyl crotonate. After quenching, the crude product mixture of **3** and **4** was isolated [12]. In spite of the fact that several attempts to separate cleanly the diastereomers were unsuccessful, we were able to determine both the reactivity and the extent of selectivity (i.e. % conversion and dr) by NMR signal integration, since the olefinic region in the spectrum is free of signals other than those of **3**, **4** and **1** (see below). It is noteworthy that NMR examination of the crude products most faithfully reflects the stereochemical outcome of the reaction.

Of the four possible diastereoisomeric conjugate addition products resulting from the formation of two new stereogenic centers (methine carbon atoms 3 and 4 of the ethyl hexenoates, see Scheme 1) only two could be detected in all cases except the 9-anthryl derivative (**3h**), which indicated a single adduct. Apart from having proceeded regioselectively (α- rather than γ- to the sulfone functionality), the addition was thus diastereoselective as well [2,13]. Both the ratio between the diastereoisomeric adducts **3** and **4** as well as the extent of reaction (% conversion) were determined by comparing the integration values for the distinct olefinic proton signals of the non-separable addition products and of any unreacted starting amino-substituted sulfone **1** [14]. The analysis of the reaction mixture for the conjugate addition of lithiated donors **1** to ethyl crotonate (**2**) is summarized in Table 2.

**Table 2:** Remote chiral induction in the conjugate addition of lithiated α-sulfonylallyl anions of **1** to ethyl crotonate (**2**).

Donor **1**	Ar	Conversion (%)	dr (3:4)^a^	Time^b^ (min)

**a**	Phenyl^c^	72	82:18	60
**b**	4-Methoxyphenyl	100	77:23	60
**c**	4-Nitrophenyl	72	86:14	120
**d**	2-Thienyl	90	82:18	60
**e**	1-Naphthyl	46	90:10	60
**f**	2-Naphthyl	100	69:31	60
**g**	2-Phenanthryl	85	81:19	120
**h**	9-Anthryl	100	>99:1	60
**i**	2-Anthryl	66	83:17	60
**j**	Mesityl	77	85:15	120

^a^Ratios determined by integration of olefinic peaks in the mixture, values ±2%, ^b^In cases of slow reactions, the time was doubled to achieve better NMR integration, ^c^[4].

By analogy with previous work on conjugate addition of α-sulfonylallylic carbanions to open-chain α,β-unsaturated esters, which has been shown to proceed *anti*-diastereoselectively [2,13] and with the *N*(1)R*,3S*,4R* relative configuration predominating [1], we assign our major and minor products structures **3** and **4** respectively (showing in Scheme 1 only one enantiomer for each racemate).

We compared the conjugate addition of lithiated carbanion **1** to crotonate **2** in which Ar of the amine segment possessed either an electron donating or withdrawing group, a heterocyclic moiety, or bicyclic and tricyclic aromatic groups (Table 2). In all cases diastereoselectivity in favor of adduct **3** was observed. Two effects can be noted. One involves the relative speed of reaction as indicated by the % conversion for a certain reaction time. The second effect is one of stereoselectivity as described by the diastereomeric ratio (dr). Substituting thiophene for phenyl (compare **1d** to **1a**) caused no change in the dr, but the reaction of the more electron rich thiophene derivative was faster. The 4-methoxyphenyl derivative **1b**, reacted faster than the 4-nitro analog **1c**, as expected due to the presence of an electron donating substituent. Surprisingly, however, there was a slight increase in diastereoselectivity for the more electron deficient nitro derivative **1c**.

**Figure 1:**
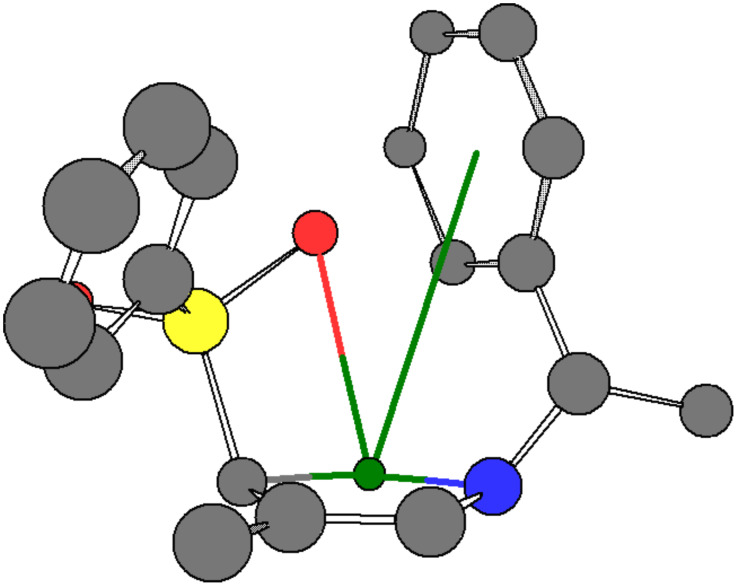
Calculated minimum energy conformation of lithiated amino-substituted sulfone **1a** showing π-interaction between Li^+^ ion and remote phenyl nucleus of the α-methylbenzylamine (1-phenylethylamine) moiety; gray = C, green = Li, blue = N, yellow = S, red = O, hydrogen atoms are omitted for clarity.

The most remarkable case of remote chirality transfer (over 99% dr), was observed for the 9-anthryl case **1h**, which showed a rate enhancement as well [15–16]. In order to determine whether an unusual steric effect may account for enhanced stereoselectivity in 9-anthryl compound **1h**, we also examined the 2-anthryl case **1i** and the mesityl derivative **1j**. In fact steric hindrance, as expected in the mesityl derivative **1j** (which resembles **1h** spatially) caused a rate retardation compared to 9-anthryl. Though the precise geometry of the aromatic ring during chelation with the lithiated species is as yet unknown, it evidently plays an important role in determining the approach of the carbanion to the conjugate acceptor and thus the stereoselectivity of the conjugate addition. Preliminary *ab initio* calculations [17] for the energetics of approach of **1** to **2** (in the case Ar = Ph) are consistent with these results: the calculations indicate that a remote aromatic substituent in lithiated **1**, in its lowest energy conformation, can come into close proximity (ca. 3.2 Å) with the lithium cation (Figure 1) [18–21]. Such an interaction between the aromatic π-system and the Li^+^ leads to stabilization.

However, the lowest energy interaction is expected to produce the *N*(1)R*,3R*,4S* configured adduct **4**. Therefore, it is the slightly less stable, but more reactive intermediary donor–acceptor complex that is apparently responsible for preferential formation of the *N*(1)R*,3S*,4R* configuration in adduct **3**.

## Conclusion

The dependence of chiral induction on the identity of the aromatic nucleus attached to a remote stereogenic center in the conjugate additions of lithiated α-(phenylsulfonyl)allylic carbanions was examined. Better yields are obtained with aromatic rings possessing higher electron density (4-methoxyphenyl, 2-thienyl) while the employment of the 9-anthryl nucleus results in complete transmission of chirality to the newly formed stereogenic centers. *Ab initio* calculations assign an important role to Li^+^–aromatic π interaction in this remote chiral control.

## Supporting Information

File 1 Experimental procedures, characterization data and NMR spectra.
