# Supplementary material for: The role of an aromatic group in remote chiral induction during conjugate addition of α-sulfonylallylic carbanions to ethyl crotonate
Source: Beilstein J Org Chem. 2008 Sep 23;4:32. doi: 10.3762/bjoc.4.32 (PMC2568876; doi:10.3762/bjoc.4.32)

**Supporting Information for**

**The role of an aromatic group in remote chiral induction during conjugate addition of α-sulfonylallylic carbanions to ethyl crotonate**

*Shlomo Levinger, Ranjeet Nair and Alfred Hassner**

*Department of Chemistry, Bar-Ilan University, Ramat-Gan 52900, Israel*

[*hassna@mail.biu.ac.il*](mailto:hassna@mail.biu.ac.il)

Experimental procedures and characterization data S2–S16

NMR spectra of amino-substituted allyl sulfones **1** and precursors S17–S36

NMR spectra of the olefinic protons of the diastereoisomeric product mixture (**3** + **4**) obtained

from the conjugate additions (**1** + **2**); representative examples S37–S38

**Experimental procedures and characterization data**

**General information:** Air- and moisture-sensitive reactions were carried out in oven- and/or flame-dried, argon-flushed flasks sealed with rubber septa, and the reagents were introduced with a syringe. Tetrahydrofuran (THF) and diethyl ether were freshly distilled from sodium/benzophenone. Flash column chromatography was done on silica gel 60 (0.040–0.063 mm, Merck) and precoated silica gel plates (60F-254, Merck) were utilized for TLC. Melting points were uncorrected. ^1^H NMR spectra were recorded at 200, 300 or 600 MHz. ^13^C NMR spectra were recorded at 50.3, 75.5 or 150.9 MHz. Signal multiplicity was determined by the DEPT technique. HMQC, HMBC and NOESY methods were used to establish atom connectivities. HRMS was based on the chemical ionization method (in methane or isobutane) unless otherwise specified.

**Preparation of primary amines 6 through Borch reductive amination of methyl ketones 7** [1]

**α-Methyl-2-naphthalenemethanamine (6f):** To a solution of 2-acetonaphthone (**7f**, 850 mg, 4.99 mmol) in methanol (13 mL) was added NH_4_OAc (6.52 g). After most of the salt has been dissolved, NaBH_3_CN (250 mg, 3.98 mmol) was added and the solution was left to stir at rt for 4 days. The reaction mixture was acidified with aq HCl (1:1, 18 mL) and rinsed with Et_2_O (2 × 20 mL). A solution of 35% aq KOH was added to the clear aq phase until the appearance of persistent turbidity and the mixture was then extracted with CH_2_Cl_2_ (3 × 40 mL) followed by drying of the extract over Na_2_SO_4_. Removal of the solvent yielded amine **6f** as a colorless oil [566.0 mg, 66% (lit. [2] 77%)]. ^1^H NMR [2] (200 MHz, CDCl_3_): *δ* 7.87–7.74 (m, 4 H; 1,4,5,8-H), 7.52–7.41 (m, 3 H; 3,6,7-H), 4.27 (q, *J* = 6.6 Hz, 1 H; C*H*CH_3_), 1.72 (br s, 2 H; N*H*_2_), 1.45 ppm (d, *J* = 6.6 Hz, 3 H; C*H*_3_).

Arylalkylamines **6b, g, i** were prepared similarly. A MeOH/CH_2_Cl_2_ (1:1) or a MeOH/CHCl_3_ (1:1) mixture was used as the reaction medium when the starting ketone showed low methanol solubility (cases **g, i**).

**α-Methyl-4-methoxybenzenemethanamine (6b):** Yield: 98% (lit. [3] 90%). ^1^H and ^13^C NMR spectra were identical to published data [3].

**α-Methyl-2-phenanthrenemethanamine (6g):** Yield: 36% (lit. [4] 60%, for the hydrochloride). ^1^H NMR (200 MHz, CDCl_3_): *δ* 8.70–8.60 (m, 2 H; 4,5-H), 7.92–7.82 (m, 2 H; 1,8-H), 7.72 (s, 2 H; 9,10-H), 7.70–7.52 (m, 3 H; 3,6,7-H), 4.33 (q, *J* = 6.7 Hz, 1 H; C*H*CH_3_), 1.70 (br s, 2 H; N*H*_2_), 1.50 (d, *J* = 6.7 Hz, 3 H; C*H*_3_); ^13^C NMR (75.5 MHz, CDCl_3_ + CD_3_OD): *δ* 143.97 (s; C-2), 131.74 (s; C-Ar), 131.53 (s; C-Ar), 129.69 (s; C-Ar), 128.97 (s; C-Ar), 128.03 (d; C-8), 126.72 (d; C-Ar), 126.31 (d; C-Ar), 126.15 (d; C-Ar), 125.99 (d; C-Ar), 124.54 (d; C-Ar), 124.08 (d; C-Ar), 122.67 (d; C-5), 122.07 (d; C-4), 50.48 (d; *C*HCH_3_), 23.97 (q; CH_3_); HRMS (DCI^+^ CH_4_): *m/z* (%) 221.120841 (28) [*M*^+^] (calc. 221.120450), 206.095104 (100) [*M*^+^ − CH_3_] (calc. 206.096974).

**α-Methyl-2-anthracenemethanamine (6i)** [5]**:** Yield: 27%. ^1^H NMR (600 MHz, CDCl_3_): δ 8.38 (s, 1 H; 9-H or 10-H), 8.37 (s, 1 H; 10-H or 9-H), 7.98-7.97 (m, 3 H; 4,5,8-H), 7.89 (s, 1 H; 1-H), 7.47 (d, *J* = 9 Hz, 1 H; 3-H), 7.43 (m, 2 H; 6,7-H), 4.29 (q, *J* = 6.6 Hz, 1 H; C*H*CH_3_), 1.69 (br s, 2 H; N*H*_2_), 1.49 (d, *J* = 6.6 Hz, 3 H; C*H*_3_); ^13^C NMR (150.9 MHz, CDCl_3_): *δ* 144.40 (s; C-2), 131.90 (s; C-8a or 10a), 131.72 (s; C-10a or 8a), 131.54 (s; C-9a), 131.09 (s; C-4a), 128.56 (d; C-4), 128.18 (d; C-5 or 8), 128.08 (d; C-8 or 5), 125.97 (d; C-9), 125.96 (d; C-10), 125.32 (d; C-6 or 7), 125.15 (d; C-7 or 6), 124.58 (d; C-3), 123.37 (d; C-1), 51.50 (d; *C*HCH_3_), 25.27 (q; CH_3_); HRMS (DCI^+^ isobutane): *m/z* (%) 221.121000 (83) [*M*^+^] (calc. 221.120450), 206.088 (100) [*M*^+^ − CH_3_], 179.090 (26) [*M*H^+^ − CH_3_CHNH_2_].

**Primary amine preparation by a modified Leuckart reaction**

**α-Methyl-2-thiophenemethanamine (6d)** [6,7]: A mixture of 2-acetylthiophene (32.14 g, 0.255 mol), formamide (49.02 g, 1.09 mol) and aqueous HCO_2_H (90%, 4 mL) was brought to reflux in a system equipped with a thermometer measuring the temperature of the vapor above the boiling liquid and a downward condenser connected to the head of the reflux condenser. In the course of 4 h the reflux temperature rose from 155 to 185 °C and the reflux condenser became clogged with ammonium carbonate. Addition of 8 mL HCO_2_H at this point freed the condenser and lowered the temperature to 165 °C. The water in the reflux condenser's jacket was drained out which resulted in rising of the temperature to 185 °C in about 1/2 h. At this point the water flow was resumed and the reflux continued at the above temperature with frequent sampling of the vapor at the head of the condenser for its pH (wet litmus paper). A few mL of HCO_2_H were added when the pH was found alkaline; this addition caused the temperature to drop down to 165 °C. This cycle of raising the reflux temperature to 185 °C by draining the condenser and lowering it to 165 °C by addition of HCO_2_H whenever the vapor pH became alkaline continued for a total of 55 h (with pausing at night); any starting ketone accumulating above the aqueous distillate in the meantime was returned to the reaction mixture.

The reaction mixture was treated carefully with 30% aq NaOH (140 mL), refluxed for 18 h (109 °C) and, after cooling, extracted with Et_2_O (5 × 100 mL). After washing the organic phase with water, a 1:1 aq HCl (220 mL) was added and the mixture was stirred vigorously for 40 min. The aqueous layer was then washed with ether and subsequently cooled in an ice bath before a cold 33% aq NaOH solution (140 mL) was slowly added with stirring, keeping the temperature below 20 °C. The alkaline solution obtained was extracted with Et_2_O (4 × 100 mL) and the extract left drying over Na_2_SO_4_. Solvent removal left a liquid residue which was distilled at 84–85 °C/18 Torr (lit. [6] 83–84 °C/16 Torr) to yield 13.3 g (41%, lit. [6] 51%) of amine **6d** as a colorless liquid. ^1^H NMR [8] (200 MHz, CDCl_3_): *δ* 7.17 (dd, *J* = 5.2, 1 Hz, 1 H; 5-H), 6.96-6.87 (m, 2 H; 3,4-H), 4.37 (qd, *J* = 6.6, 0.5 Hz, 1 H; C*H*CH_3_), 1.77 (br s, 2 H; N*H*_2_), 1.48 (d, *J* = 6.6 Hz, 3 H; C*H*_3_).

**Preparation of the (9-anthryl)-based primary amine 6h**

**α-Methyl-9-anthracenemethanimine (8h)** [9]**:** MeMgI was prepared by the usual method from Mg granules (125 mg, 5.14 mmol) and iodomethane (710 mg, 5.00 mmol) in Et_2_O (1 mL) under argon. Benzene (6.5 mL) was injected and the solvent mixture was distilled down to a volume of 3 mL by which process the boiling temperature had been raised to 70 °C. The mixture was cooled down to rt and 4 mL benzene were added followed by 9-anthracenecarbonitrile (1.02 g, 5.00 mmol). The reaction mixture was heated under reflux (78 °C) for 1.5 h and after cooling down to 4 °C was added in portions and with stirring to an ice-cold, saturated aqueous NH_4_Cl (1.5 mL). The aqueous layer was washed with ether and the combined organic phase was dried over Na_2_SO_4_. The residue after solvent evaporation was purified by silica gel column chromatography (77 g, hexane/EtOAc 1.5:1) to yield imine **8h** (505 mg, 46%) which was used in the reduction to the amine without further purification. ^1^H NMR (300 MHz, CDCl_3_): *δ* 8.39 (br s, 1 H; 10-H), 7.98 (m, 2 H; 4,5-H), 7.89 (m, 2 H; 1,8-H), 7.48 (m, 2 H; 2,7-H), 7.46 (m, 2 H; 3,6-H), 2.60 (s, 3 H; CH_3_); ^13^C NMR (75.5 MHz, CDCl_3_): *δ* 180.50 (s; C=NH), 137.26 (s; C-9), 131.20 (s; C-4a,10a), 128.67 (d; C-4,5), 127.13 (d; C-10), 126.53 (s; C-8a,9a), 126.37 (d; C-2,7), 125.42 (d; C-3,6), 124.88 (d; C-1,8), 29.09 (q; CH_3_).

**α-Methyl-9-anthracenemethanamine (6h)** [10]**:** A solution of NaBH_3_CN (728 mg, 11.6 mmol) in methanol (3 mL) was added to a stirring solution of imine **8h** (505 mg, 2.30 mmol) in dichloromethane (7 mL) followed by addition of glacial acetic acid (0.8 mL). After standing for 3 d at rt, 5% aqueous KOH (17 mL) was added, the organic layer was separated out and the aqueous phase was extracted with CH_2_Cl_2_ (2 × 15 mL). The combined organic phase was washed with water and left to dry over Na_2_SO_4_. Solvent evaporation yielded 472 mg of the amine **6h** (93%, lit. [10] 96%). ^1^H NMR [10] (200 MHz, CDCl_3_): *δ* 8.79–8.61 (br coalescing two peak signal, 2 H; 1,8-H), 8.33 (br s, 1 H; 10-H), 7.99 (m, 2 H; 4,5-H), 7.55-7.38 (m, 4 H; 2,3,6,7-H), 5.75 (q, *J* = 7.0 Hz, 1 H; C*H*CH_3_), 2.07 (br s, 2 H; N*H*_2_), 1.83 (d, *J* = 7.0 Hz, 3 H; C*H*_3_); ^13^C NMR [10] (50.3 MHz, CDCl_3_): *δ* 137.76, 131.96, 129.46, 127.32, 125.57, 125.18 (br), 124.68, 46.54, 24.12.

Note: The broad proton signal at δ 8.79–8.61 indicates an ongoing dynamic process which we ascribe to restricted rotation around the anthryl-side chain bond; this resonance is not evidenced in the literature NMR data for this compound [10].

**Preparation of the mesityl-based primary amine 6j**

**2,4,6-Trimethylbenzenecarbonitrile (mesityl cyanide):** Prepared in 89% yield according to published procedure [11]**.** ^1^H and ^13^C NMR spectra were identical with documented data [12].

**α,2,4,6-Tetramethylbenzenemethanimine (8j)** [13]: To MeMgI, prepared in the usual way from Mg (1.45 g, 59.6 mmol) and iodomethane (8.52 g, 60.0 mmol) in ether (14 mL), was slowly added a solution of mesityl cyanide (4.35 g, 30.0 mmol) in dry benzene (20 mL), after which the mixture was refluxed for 3 h. The reaction progress was monitored by TLC (hexane/EtOAc 8:2). The reaction mixture was cooled down to 4 °C, quenched by adding it to an ice-cold, saturated NH_4_Cl and divided between water and CH_2_Cl_2_ (15 mL each). The organic layer was dried over Na_2_SO_4_ and the residue after solvent evaporation was purified by silica gel column chromatography (hexane/EtOAc 9:1) to yield imine **8j** (3.0 g, 62%) showing ^1^H NMR spectrum identical to lit. data [13].

**α,2,4,6-Tetramethylbenzenemethanamine (6j)** [13]**:** Imine **8j** (2.42 g, 15.0 mmol) was dissolved, under argon atmosphere, in acetonitrile (10 mL). To this stirred solution was added sodium cyanoborohydride (9.45 g, 151 mmol) dissolved in 10 mL of acetonitrile followed by boric acid (7.26 g, 117 mmol). Glacial acetic acid (3 mL) dissolved in 3 mL of acetonitrile was slowly added to the reaction mixture followed by another dose of the AcOH/MeCN mixture after 2 h. The reaction progress was monitored by TLC (hexane/EtOAc, 1:4). After quenching with 20 mL of 60% NaOH, the reaction mixture was extracted with 30 mL CHCl_3_. The organic layer was washed with saturated aqueous NaHCO_3_ followed by brine and then dried over Na_2_SO_4_. Solvent evaporation yielded the amine **6j** as an oil (1.9 g, 78%) which was used for the next step without further purification. ^1^H NMR was identical with published data [13].

**Preparation of α-(phenylsulfonyl)allylic donor precursors 1**

**General procedure** [14]: To a stirred solution of primary amine **6** (1.6 mmol) in the presence of K_2_CO_3_ (420 mg, 3.04 mmol) in DMF (6 mL) and under argon flow, was added dropwise a solution of bromo-substituted sulfone **5** [15] (1.0 mmol) in the same solvent (5 mL) at 0 °C. Stirring of the reaction mixture was continued at rt while the consumption of **5** was monitored by TLC (hexane/EtOAc, 3:1). At the end of the reaction (1–3 d), the mixture was diluted with water (30 mL) and extracted with Et_2_O/CH_2_Cl_2_ (4:1, 3 × 25 mL). The organic extract was thoroughly washed with water (9 × 30 mL) to remove traces of DMF, dried over Na_2_SO_4_, filtered and evaporated. Column chromatography of the residue over silica gel (ratio of silica to the crude mixture and eluent composition are given separately for each product) afforded the pure amino-substituted sulfone **1**.

***N*-[2-[(Phenylsulfonyl)methyl]prop-2-enyl]-α-methyl-4-methoxybenzenemethanamine (1b)**: 68 g Silica/1 g crude; eluent: EtOAc; yield: 92%; mp 60–61 °C (Et_2_O); ^1^H NMR (200 MHz, CDCl_3_): *δ* 7.82 (m, 2 H; *o*-PhS(O_2_)), 7.61 (m, 1 H; *p*-PhS(O_2_)), 7.51 (m, 2 H; *m*-PhS(O_2_)), 7.17 (d, *J* = 9.0 Hz, 2 H; 2-*H* (MeOC_6_H_4_)), 6.85 (d, *J* = 9.0 Hz, 2 H; 3-*H* (MeOC_6_H_4_)), 5.20 (q, *J* = 1 Hz, 1 H; olefinic), 4.91 (br s, 1 H; olefinic), 3.89 (br s, 2 H; C*H*_2_(O_2_)S), 3.80 (s, 3 H; OMe), 3.62 (q, *J* = 6.6 Hz, 1 H; C*H*CH_3_), 3.11 and 3.03 (AB q, *J*_gem_ = 14.2 Hz, 2 H; C*H*_2_NH), 1.61 (br s, 1 H; NH), 1.28 ppm (d, *J* = 6.6 Hz, 3 H; CHC*H*_3_); ^13^C NMR (50.3 MHz, CDCl_3_): *δ* 158.64 (s; C-4 (MeOC_6_H_4_)), 138.70 (s; *ipso*-PhS(O_2_)), 137.43 (s; C-1 (MeOC_6_H_4_)), 136.33 (s; olefinic), 133.57 (d; *p*-PhS(O_2_)), 128.96 (d; *m*-PhS(O_2_)), 128.50 (d; *o*-PhS(O_2_)), 127.59 (d; C-2 (MeOC_6_H_4_)), 120.44 (t; olefinic), 113.86 (d; C-3 (MeOC_6_H_4_)), 60.37 (t; CH_2_(O_2_)S), 56.86 (d *C*HCH_3_), 55.28 (q OMe), 51.78 (t; CH_2_NH), 24.20 ppm (q; CH*C*H_3_); HRMS (DCI^+^ CH_4_): *m/z* (%): 346.144900 (79) [*M*^+^ − H] (calcd: 346.147691).

**(*S*)**-***N*-[2-[(Phenylsulfonyl)methyl]prop-2-enyl]-α-methyl-4-nitrobenzenemethanamine (1c):** The (*S*)-form of the primary amine (**6c**) hydrochloride was used as reactant. Column chromatography of the crude product: 94 g silica/1 g crude; eluent: EtOAc; yield: 68%; mp 68–69 °C (Et_2_O); ^1^H NMR (200 MHz, CDCl_3_): *δ* 8.19 (d, *J* = 9.0 Hz, 2 H; 3-*H* (NO_2_C_6_H_4_)), 7.84 (m, 2 H; *o*-PhS(O_2_)), 7.62 (m, 1 H; *p*-PhS(O_2_)), 7.58-7.42 (m, 4 H; *m*-PhS(O_2_) and 2-*H* (NO_2_C_6_H_4_)), 5.23 (q, *J* = 1 Hz, 1 H; olefinic), 4.90 (br s, 1 H; olefinic), 3.90 (s, 2 H; C*H*_2_(O_2_)S), 3.85 (q, *J* = 7.1 Hz, 1 H; C*H*CH_3_), 3.20 and 3.10 (AB q, *J*_gem_ = 14.2 Hz, 2 H; C*H*_2_NH), 1.79 (br s, 1 H; NH), 1.36 (d, *J* = 7.1 Hz, 3 H; CHC*H*_3_); ^13^C NMR (75.5 MHz, CDCl_3_): *δ* 153.16 (s; C-1 (NO_2_C_6_H_4_)), 147.11 (s; C-4 (NO_2_C_6_H_4_)), 138.48 (s; *ipso*-PhS(O_2_)), 135.67 (s; olefinic), 133.76 (d; *p*-PhS(O_2_)), 129.06 (d; *m*-PhS(O_2_)), 128.42 (d; *o*-PhS(O_2_)), 127.48 (d; C-2 (NO_2_C_6_H_4_)), 123.82 (d; C-3 (NO_2_C_6_H_4_)), 121.06 (t; olefinic), 60.42 (t; CH_2_(O_2_)S), 57.24 (d; *C*HCH_3_), 51.88 (t; CH_2_NH), 24.25 (q; CH_3_); HRMS (DCI^+^ CH_4_): *m/z* (%): 361.121826 (59) [*M*H^+^] (calc. 361.122204).

***N*-[2-[(Phenylsulfonyl)methyl]prop-2-enyl]-α-methyl-2-thiophenemethanamine (1d):** 174 g Silica/1 g crude; eluent: hexane/EtOAc (4:5); yield: 48%; mp 64–65 °C (Et_2_O); ^1^H NMR (200 MHz, CDCl_3_): *δ* 7.85 (m, 2 H; *o*-PhS(O_2_)), 7.60 (m, 1 H; *p*-PhS(O_2_)), 7.51 (m, 2 H; *m*-PhS(O_2_)), 7.19 (dd, *J* = 5, 1 Hz, 1 H; 5-H (thienyl)), 6.92 (dd, *J* = 5, 4 Hz, 1 H; 4-H (thienyl)), 6.85 (dd, *J* = 4, 1 Hz, 1 H; 3-H (thienyl)), 5.24 (q, *J* = 1.5 Hz, 1 H; olefinic), 4.97 (q, *J* = 0.5 Hz, 1 H; olefinic), 3.97 (q, *J* = 6.2 Hz, 1 H; C*H*CH_3_), 3.93 (br s, 2 H; C*H*_2_(O_2_)S), 3.22 and 3.15 (AB q, *J*_gem_ = 14.2 Hz, 2 H; C*H*_2_NH), 1.73 (br s, 1 H; NH), 1.42 (d, *J* = 6.2 Hz, 3 H; CHC*H*_3_); ^13^C NMR (75.5 MHz, CDCl_3_): *δ* 150.26 (s; C-2 (thienyl)), 138.54 (s; *ipso*-PhS(O_2_)), 135.86 (s; olefinic), 133.60 (d; *p*-PhS(O_2_)), 128.96 (d; *m*-PhS(O_2_)), 128.48 (d; *o*-PhS(O_2_)), 126.44 (d; C-4 (thienyl)), 123.71 (d; C-5 or C-3 (thienyl)), 123.49 (d; C-3 or C-5 (thienyl)), 120.91 (t; olefinic), 60.12 (t; CH_2_(O_2_)S), 52.85 (d; *C*HCH_3_), 51.43 (t; CH_2_NH), 24.44 (q; CH_3_); HRMS (DCI^+^ CH_4_) *m/z* (%): 322.092674 (37) [*M*H^+^] (calc. 322.093548).

***N*-[2-[(Phenylsulfonyl)methyl]prop-2-enyl]-α-methyl-1-naphthalenemethanamine (1e):** 82 g Silica/1 g crude; eluent: hexane/EtOAc (3:2); yield: 100%; oil; ^1^H NMR spectrum identical with the one already published (together with ^13^C NMR and MS) [14].

***N*-[2-[(Phenylsulfonyl)methyl]prop-2-enyl]-α-methyl-2-naphthalenemethanamine (1f):** 95 g Silica/1 g crude; eluent: hexane/EtOAc (3:2); yield: 63%; mp 73–74 °C (Et_2_O); ^1^H NMR (300 MHz, CDCl_3_): *δ* 7.85–7.74 (m, 5 H; *o*-PhS(O_2_) and 4,5,8-H (naphthyl)), 7.64 (br s, 1 H; 1-H (naphthyl)), 7.50 (tt, *J* = 8, 1.5 Hz, 1 H; *p*-PhS(O_2_)), 7.49-7.34 (m, 5 H; *m*-PhS(O_2_) and 3,6,7-H (naphthyl)), 5.22 (q, *J* = 1.3 Hz, 1 H; olefinic), 4.95 (q, *J* = 0.6 Hz, 1 H; olefinic), 3.90 (AB q, *J*_gem_ = 14 Hz, 2 H; C*H*_2_(O_2_)S), 3.83 (q, *J* = 6.6 Hz, 1 H; C*H*CH_3_), 3.14 and 2.98 (AB q, *J*_gem_ = 14.4 Hz, 2 H; C*H*_2_NH), 1.40 (d, *J* = 6.6 Hz, 3 H; CHC*H*_3_); ^13^C NMR (75.5 MHz, CDCl_3_): *δ* 142.58 (s; C-2 (naphth)), 138.40 (s; *ipso*-PhS(O_2_)), 136.08 (s; olefinic), 133.44 (d; *p*-PhS(O_2_)), 133.38 (s; C-4a (naphth)), 132.79 (s; C-8a (naphth)), 128.81 (d; *m*-PhS(O_2_)), 128.34 (d; *o*-PhS(O_2_)), 128.22 (d; naphth), 127.66 (d; naphth), 127.59 (d; naphth), 125.95 (d; naphth), 125.48 (d; naphth), 125.27 (d; naphth), 124.67 (d; naphth), 120.58 (t; olefinic), 60.24 (t; CH_2_(O_2_)S), 57.59 (d; *C*HCH_3_), 51.78 (t; CH_2_NH), 24.11(q; CH_3_); HRMS (DCI^+^ CH_4_) *m/z* (%): 366.145058 (1) [*M*H^+^] (calc. 366.152776), 350.1183 (52) [*M*H^+^ − H − CH_3_], 224.1393 (24) [*M*H^+^ − PhS(O)OH], 155.0811 (58) [C_10_H_7_CHCH_3_^+^].

***N*-[2-[(Phenylsulfonyl)methyl]prop-2-enyl]-α-methyl-2-phenanthrenemethanamine (1g):** 78 g Silica/1 g crude; eluent: hexane/EtOAc (2:1); yield: 63%; oil; ^1^H NMR (200 MHz, CDCl_3_): *δ* 8.70–8.60 (m, 2 H; 4,5-H (phenanthryl)), 7.90–7.32 (m, 12 H; PhS(O_2_) and 1,3,6,7,8,9,10-H (phenanthryl)), 5.24 (br s, 1 H; olefinic), 4.98 ((br s, 1 H; olefinic), 3.93 (br s, 2 H; C*H*_2_(O_2_)S), 3.91 (q, *J* = 6.7 Hz, 1 H; C*H*CH_3_), 3.18 and 3.02 (AB q, *J*_gem_ = 14.0 Hz, 2 H; C*H*_2_NH), 2.0 (br s, 1 H; NH), 1.45 (d, *J* = 6.7 Hz, 3 H; CHC*H*_3_); ^13^C NMR (75.5 MHz, CDCl_3_): *δ* 143.06 (s; C-2 (phenanth)), 138.32 (s; *ipso*-PhS(O_2_)), 135.62 (s; olefinic), 133.50 (d; *p*-PhS(O_2_)), 132.04 (s; C-4a or C-4b (phenanth)), 131.87 (s; C-4b or C-4a (phenanth)), 130.17 (s; C-8a or C-10a (phenanth)), 129.46 (s; C-10a or C-8a (phenanth)), 128.85 (d; *m*-PhS(O_2_)), 128.54 (d; C-8 (phenanth)), 128.34 (d; *o*-PhS(O_2_)), 127.09 (d; phenanth), 126.83 (d; phenanth), 126.61 (d; phenanth), 126.41 (d; phenanth), 126.33 (d; phenanth), 125.24 (d; phenanth), 123.05 (d; C-5 (phenanth)), 122.54 (d; C-4 (phenanth)), 121.17 (t; olefinic), 60.25 (t; CH_2_(O_2_)S), 57.49 (d; *C*HCH_3_), 51.71 (t; CH_2_NH), 24.12 (q; CH_3_); HRMS (DCI^+^ CH_4_) *m/z* (%): 416.168058 (30) [*M*H^+^] (calc. 416.168426).

***N*-[2-[(Phenylsulfonyl)methyl]prop-2-enyl]-α-methyl-9-anthracenemethanamine (1h):** 130 g Silica/1 g crude; eluent: hexane/EtOAc (3:2); yield: 80%; oil; ^1^H NMR (200 MHz, CDCl_3_): *δ* 8.37 (br s, 1 H; 10-H (anthryl)), 8.00 (m, 2 H; 4,5-H (anthryl)), 7.58 (m, 2 H; *o*-PhS(O_2_)), 7.46-7.35 (m, 5 H; *p*-PhS(O_2_) and 2,3,6,7-H (anthryl)), 7.28 (m, 2 H; *m*-PhS(O_2_)), 5.36 (q, *J* = 7.4 Hz, 1 H; C*H*CH_3_), 5.14 (q, *J* = 1.5 Hz, 1 H; olefinic), 4.93 (br s, 1 H; olefinic), 3.82 and 3.76 (AB q, *J*_gem_ = 13 Hz, 2 H; C*H*_2_(O_2_)S), 3.23 and 2.75 (AB q, *J*_gem_ = 14 Hz, 2 H; C*H*_2_NH), 1.72 (d, *J* = 7.4 Hz, 4 H; CHC*H*_3_ and NH); ^13^C NMR (75.5 MHz, CDCl_3_): *δ* 138.37 (s; *ipso*-PhS(O_2_)), 136.21 (s; olefinic or C-9 (anthr)),135.29 (s; C-9 (anthr) or olefinic), 133.26 (d; *p*-PhS(O_2_)), 131.76 (br; anthr), 129.69 (br; anthr), 129.31 (d; C-4,5 (anthr)), 128.69 (d; *m*-PhS(O_2_)), 128.21 (d; *o*-PhS(O_2_)), 127.44 (d; C-10 (anthr)), ~125 (br; anthr), 124.69 (d; C-3,6 (anthr)), ~123 (br; anthr), 120.70 (t; olefinic), 59.99 (t; CH_2_(O_2_)S), 53.18 (t; CH_2_NH), 53.01 (d; *C*HCH_3_), 22.18 (q; CH_3_); HRMS (DCI^+^ CH_4_) *m/z* (%): 416.167839 (6) [*M*H^+^] (calc. 416.168426), 274.160484 (100) [*M*H^+^ − PhS(O)OH] (calc. 274.159575).

Note: The shallow broadenings around *δ* 9.2 and 8.4 ppm in the ^1^H NMR spectrum are apparently due to protons 1 and 8 of the anthryl nucleus exhibiting distinctive resonances owing to restricted rotation around the anthryl-side chain bond; the broad resonances in the ^13^C NMR spectrum are presumably evidence for the same ongoing dynamic process.

***N*-[2-[(Phenylsulfonyl)methyl]prop-2-enyl]-α-methyl-2-anthracenemethanamine (1i)**: 130 g Silica/1 g crude; eluent: hexane/EtOAc (1:1); yield: 59%; mp 143–145 °C (chloroform/hexane); ^1^H NMR (600 MHz, CDCl_3_): *δ* 8.40 (s, 1 H; 10-H (anthryl)), 8.36 (s, 1 H; 9-H (anthryl)), 8.00 (m, 2 H; 5,8-H (anthryl)), 7.98 (d, *J* = 9.0 Hz, 1 H; 4-H (anthryl)), 7.77 (br s, 1 H; 1-H (anthryl)), 7.75 (dd, *J* = 8.1, 1 Hz, 2 H; *o*-PhS(O_2_)), 7.47 (m, 1 H; *p*-PhS(O_2_)), 7.45 (m, 2 H; 6,7-H (anthryl)), 7.43 (dd, *J* = 8.7, 1.5 Hz, 1 H; 3-H (anthryl)), 7.33 (t, *J* = 7.8 Hz, 2 H; *m*-PhS(O_2_)), 5.23 (s, 1 H; olefinic), 4.96 (s, 1 H; olefinic), 3.92 and 3.90 (AB q, *J*_gem_ = 13.8 Hz, 2 H; C*H*_2_(O_2_)S), 3.86 (q, *J* = 6.6 Hz, 1 H; C*H*CH_3_), 3.18 and 3.02 (AB q, *J*_gem_ = 14 Hz, 2 H; C*H*_2_NH), 1.75 (br s, 1 H; NH), 1.42 (d, *J* = 6.6 Hz, 3 H; CHC*H*_3_); ^13^C NMR (150.9 MHz, CDCl_3_): *δ* 141.68 (s; C-2 (anthr)), 138.39 (s; *ipso*-PhS(O_2_)), 135.97 (s; olefinic), 133.48 (d; *p*-PhS(O_2_)), 131.85 (s; C-8a (anthr)), 131.57 (s; C-10a or C-4a (anthr)), 131.54 (s; C-4a or C-10a (anthr)), 131.24 (s; C-9a (anthr)), 128.84 (d; *m*-PhS(O_2_)), 128.67 (d; C-4 (anthr)), 128.38 (d; *o*-PhS(O_2_)), 128.15 (s; C-5 or C-8 (anthr)), 128.04 (s; C-8 or C-5 (anthr)), 125.96 (d; C-10 (anthr)), 125.85 (d; C-9 (anthr)), 125.36 (d; C-1 (anthr)), 125.22 (s; C-6 or C-7 (anthr)), 125.19 (s; C-7 or C-6 (anthr)), 124.47 (d; C-3 (anthr)), 120.83 (t; olefinic), 60.26 (t; CH_2_(O_2_)S), 57.73 (d; *C*HCH_3_), 51.81 (t; CH_2_NH), 23.72 (q; CH_3_); HRMS (FAB^+^ nba) *m/z* (%): 416.172999 (43) [*M*H^+^] (calc. 416.168426), 400.226 (17) [*M*H^+^ − H − CH_3_], 205.201 (100) [C_14_H_9_CHCH_3_^+^].

***N*-[2-[(Phenylsulfonyl)methyl]prop-2-enyl]-α,2,4,6-tetramethylbenzenemethanamine** **(1j):** Eluent: hexane/EtOAc (3:7); yield: 40%; oil; ^1^H NMR (300 MHz, CDCl_3_): *δ* 7.82 (m, 2 H; *o*-PhS(O_2_)), 7.63–7.47 (m, 3 H; *p*- and *m*-PhS(O_2_)), 6.78 (s, 2 H; mesityl), 5.19 (s, 1 H; olefinic), 4.91 (s, 1 H; olefinic), 4.16 (q, *J* = 6.9 Hz, 1 H; C*H*CH_3_), 3.89 (s, 2 H; C*H*_2_(O_2_)S), 3.11 and 2.97 (AB q, *J*_gem_ = 12.9 Hz, 2 H; C*H*_2_NH), 2.36 (s, 6 H; mesityl), 2.24 (s, 3 H; mesityl), 1.33 (d, *J* = 6.9 Hz, 3 H; CHC*H*_3_).

**Conjugate additions of amino-substituted allyl sulfones 1 to ethyl crotonate**

**General procedure** [14]**:** To a solution of amino-substituted sulfone **1** (1 mmol) in THF (5 mL), stirred under argon at −78 °C, was slowly added a solution of LDA, prepared at 0 °C from diisopropylamine (0.21 mL, 1.5 mmol) and *n*-BuLi (0.87 mL, 1.6 m in hexane, 1.4 mmol) in THF (6 mL). 40 min later, a solution of ethyl crotonate (**2**, 0.16 mL, 1.3 mmol) in THF (3 mL) was added dropwise. Stirring was continued at the above temperature for a measured period of time (see table in the text) after which the reaction mixture was quenched with saturated aqueous NH_4_Cl (2 mL). Water was added and the mixture was extracted with CH_2_Cl_2_. The organic extract was washed with aqueous NaHCO_3_ followed by brine, dried over Na_2_SO_4_, filtered and evaporated. The olefinic region in the ^1^H NMR spectrum of the crude product mixture was examined for the diastereoisomeric ratio of the conjugate adducts and for the extent of the reaction.

**1b** + **2:** ^1^H NMR (200 MHz, CDCl_3_): **major adduct:** *δ* 5.52 (br s, rel integration 1), 5.37 (br s, 0.99); **minor adduct:** *δ* 5.45 (br s, 0.33), 5.34 (br s, 0.26). HRMS (DCI^+^ CH_4_) *m/z* (%): 460.215000 (7) [adduct *M*H^+^] (calc. 460.215770).

**1c** + **2:** ^1^H NMR (200 MHz, CDCl_3_): **major adduct:** *δ* 5.55 (br s, rel integration 1), 5.42 (br s, 1.16); **minor adduct:** *δ* 5.48 (br s, 0.16), 5.42 (br s, 0.16); **1c:** *δ* 5.22 (q, *J* = 1 Hz, 0.45), 4.90 (br s, 0.45). HRMS (DCI^+^ CH_4_) *m/z* (%): 475.190498 (18) [adduct *M*H^+^] (calc. 475.190284).

**1d** + **2:** ^1^H NMR (600 MHz, CDCl_3_): **major adduct:** *δ* 5.56 (br s, rel integration 1), 5.41 (br s, 1.00); **minor adduct:** *δ* 5.47 (br s, 0.22), 5.37 (br s, 0.22); **1d:** *δ*=5.24 (br s, 0.14), 4.97 (br s, 0.13). HRMS (DCI^+^ CH_4_): *m/z* (%): 436.160000 (75) [adduct *M*^+^ − H] (calcd: 436.161627).

**1e** + **2:** ^1^H NMR (200 MHz, CDCl_3_): **major adduct:** *δ* 5.56 (br s, rel integration 1), 5.41 (br s, 1.0); **minor adduct:** *δ* 5.47 (br s, 0.11), 5.41 (br s, 0.10); **1e:** *δ* 5.22 (q, *J* = 1 Hz, 1.30), 4.95 (br s, 1.32).

**1f** + **2:** ^1^H NMR (200 MHz, CDCl_3_): **major adduct:** *δ* 5.56 (br s, rel integration 1), 5.42 (br s, 1.05); **minor adduct:** *δ* 5.47 (br s, 0.54), 5.38 (br s, 0.41). HRMS (DCI^+^ CH_4_) *m/z* (%): 480.219131 (51) [adduct *M*H^+^] (calc. 480.220856).

**1g** + **2:** ^1^H NMR (200 MHz, CDCl_3_): **major adduct:** *δ* 5.58 (br s, rel integration 1), 5.42 (br s, 1.02); **minor adduct:** *δ* 5.49 (br s, 0.22), 5.40 (br s, 0.23); **1g:** *δ* 5.23 (q, *J* = 1 Hz, 0.22), 4.97 (br s, 0.22). HRMS (DCI^+^ CH_4_) *m/z* (%): 530.237925 (3) [adduct *M*H^+^] (calc. 530.236506).

**1h** + **2:** ^1^H NMR (600 MHz, CDCl_3_): *δ* 5.53 (br s, rel integration 1), 5.37 (br s, 1.00).

**1i** + **2:** ^1^H NMR (300 MHz, CDCl_3_): **major adduct:** *δ* 5.56 (br s, rel integration 1), 5.42 (br s, 0.94); **minor adduct:** *δ* 5.47 (br s, 0.20), 5.37 (br s, 0.20); **1i:** *δ* 5.23 (br s, 0.64), 4.95 (br s, 0.61). HRMS (DCI^+^ CH_4_) *m/z* (%): 530.236404 (2) [adduct *M*H^+^] (calc. 530.236506).

**1j** + **2:** ^1^H NMR (300 MHz, CDCl_3_): **major adduct:** *δ* 5.50 (br s, rel integration 1), 5.35 (br s, 1.12); **minor adduct:** *δ* 5.42 (br s, 0.16), 5.30 (br s, 0.22); **1j:** *δ* 5.16 (q, *J* = 1 Hz, 0.36), 4.88 (br s, 0.40). HRMS (DCI^+^ CH_4_) *m/z* (%): 472.247869 (8) [adduct *M*H^+^] (calc. 472.252156).

**References**

1. Kinbara, K.; Harada, Y.; Saigo, K. *J. Chem. Soc., Perkin Trans. 2* **2000**, 1339–1347.
2. Guel, N.; Nelson, J. H. *J. Mol. Struct.* **1999**, *475*, 121–130.
3. Hose, D. R. J.; Mahon, M. F.; Molloy, K. C.; Raynham, T.; Wills, M. *J. Chem. Soc., Perkin Trans. 1* **1996**, 691–703.
4. Grisar, J. M.; Claxton, G. P.; MacKenzie, R. D. *J. Med. Chem.* **1976**, *19*, 503–508.
5. Goto, J.; Ito, M.; Katsuki, S.; Saito, N.; Nambara, T. *J. Liq. Chromatogr.* **1986**, *9*, 683–694.
6. Blicke, F. F.; Burckhalter, J. H. *J. Am. Chem. Soc.* **1942**, *64*, 477–480.
7. Moore, M. L. *Org. React.* **1949**, *5*, 301–330.
8. Viallon, L.; Reinaud, O.; Capdevielle, P.; Maumy, M. *Tetrahedron* **1996**, *52*, 7841–7854.
9. Martynoff, M.; Chauvin, M.; Grumez, M.; Lefèvre, N. *Bull. Soc. Chim. Fr.* **1958**, 164–173.
10. Kuehn, M.; Buddrus, J. *Tetrahedron: Asymmetry* **1993**, *4*, 207–210.
11. Gore, P. H.; Kamounah, F. S.; Miri, A. Y. *Tetrahedron* **1979**, *35*, 2927–2929.
12. Fergus, S.; Eustace, S. J.; Hegarty, A. F. *J. Org. Chem.* **2004**, *69*, 4663–4669.
13. Kohara, T.; Hashimoto, Y.; Shioya, R.; Saigo, K. *Tetrahedron: Asymmetry* **1999**, *10*, 4831–4840.
14. Ghera, E.; Kleiman, V.; Hassner, A. *J. Org. Chem.* **1999**, *64*, 8–9.
15. Ghera, E.; Yechezkel, T.; Hassner, A. *J. Org. Chem.* **1996**, *61*, 4959–4966.


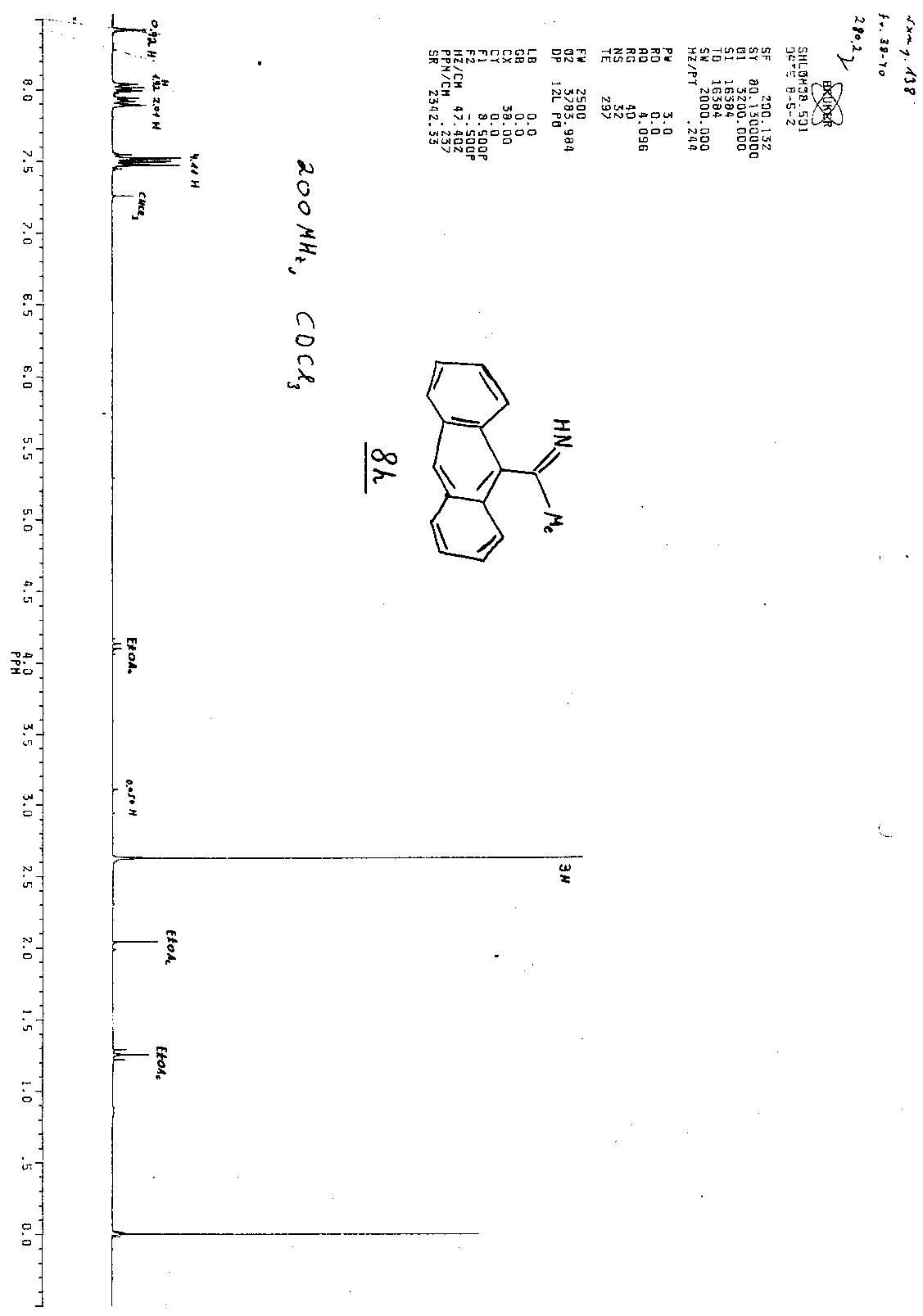

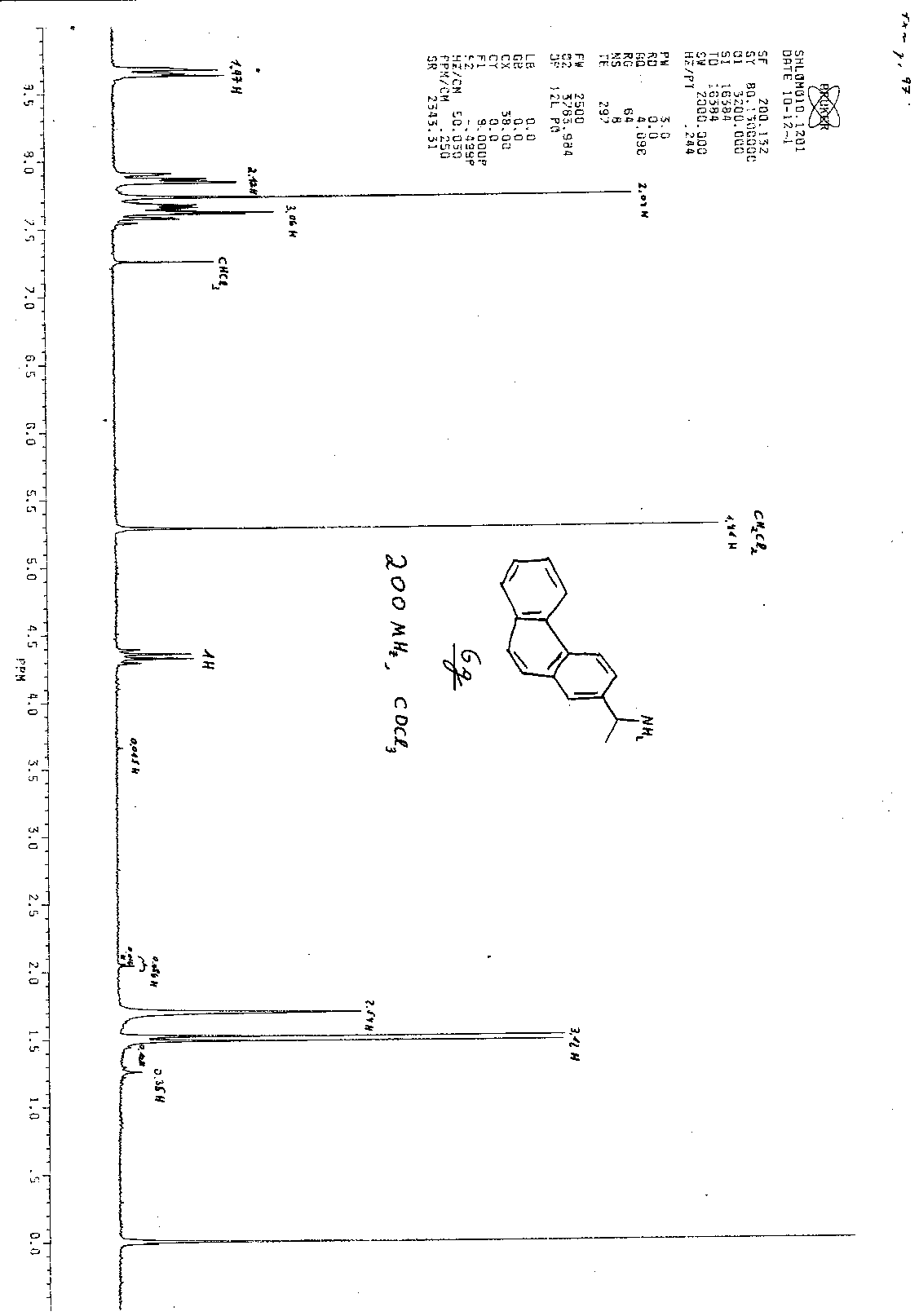

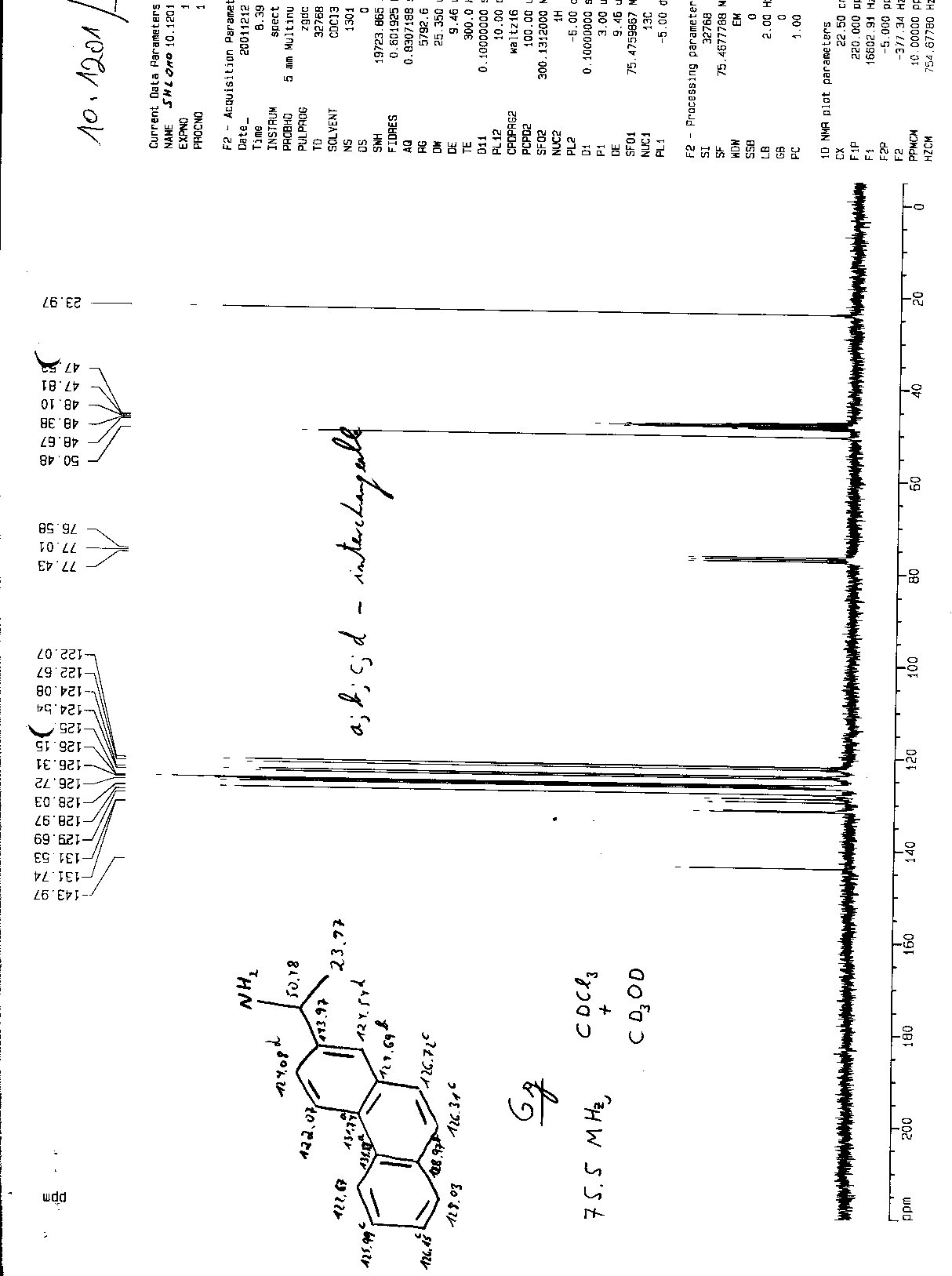

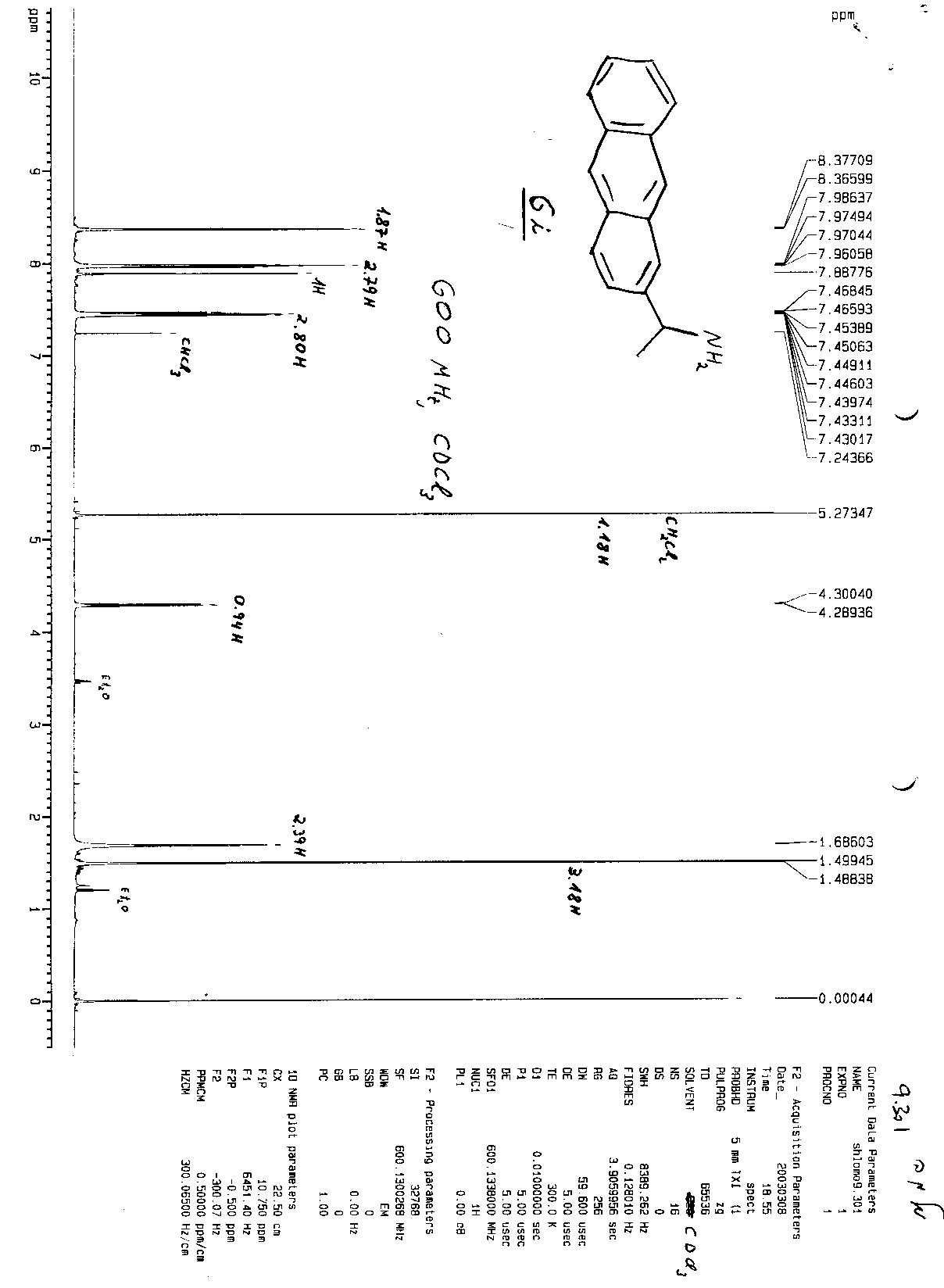

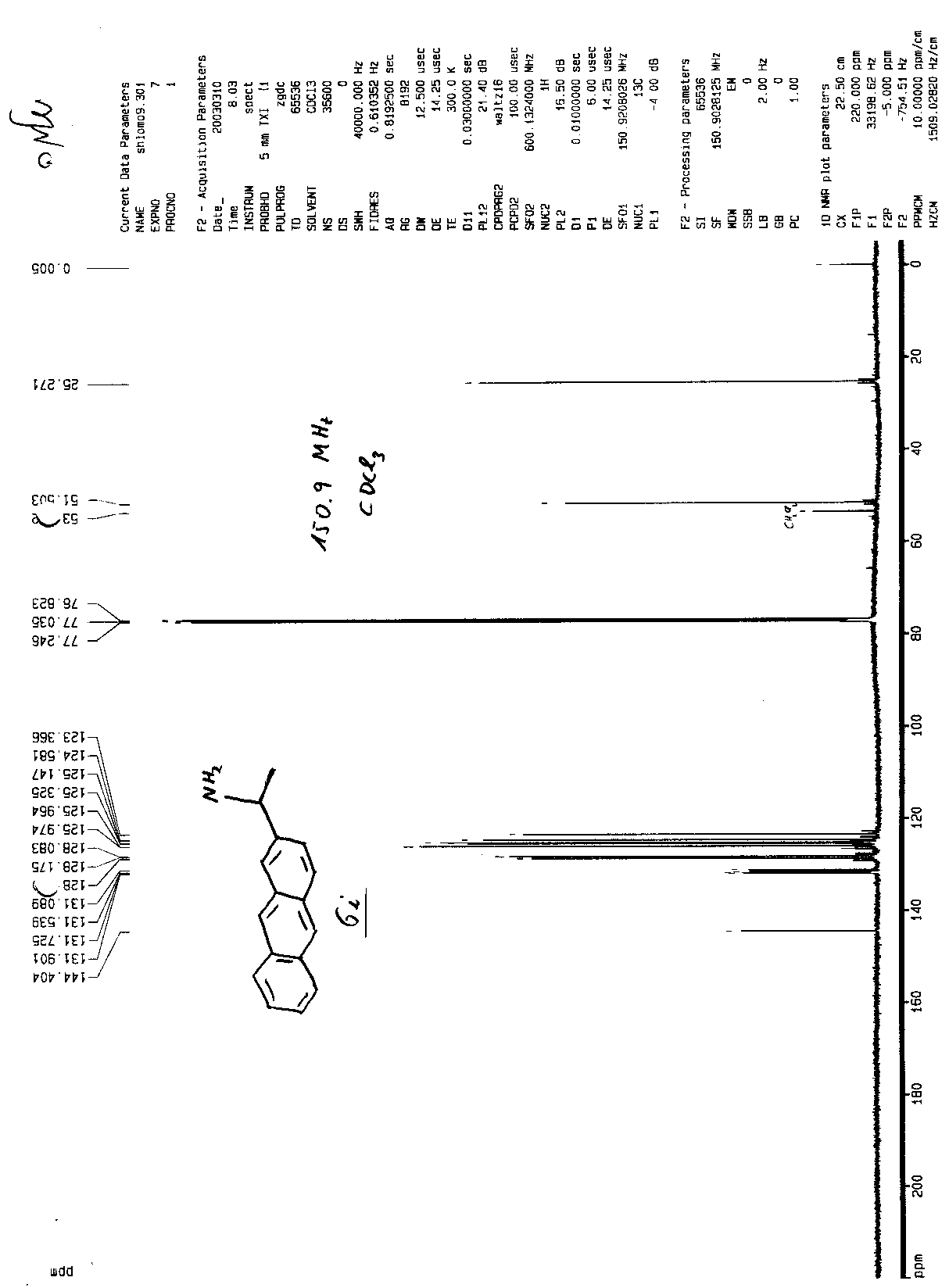

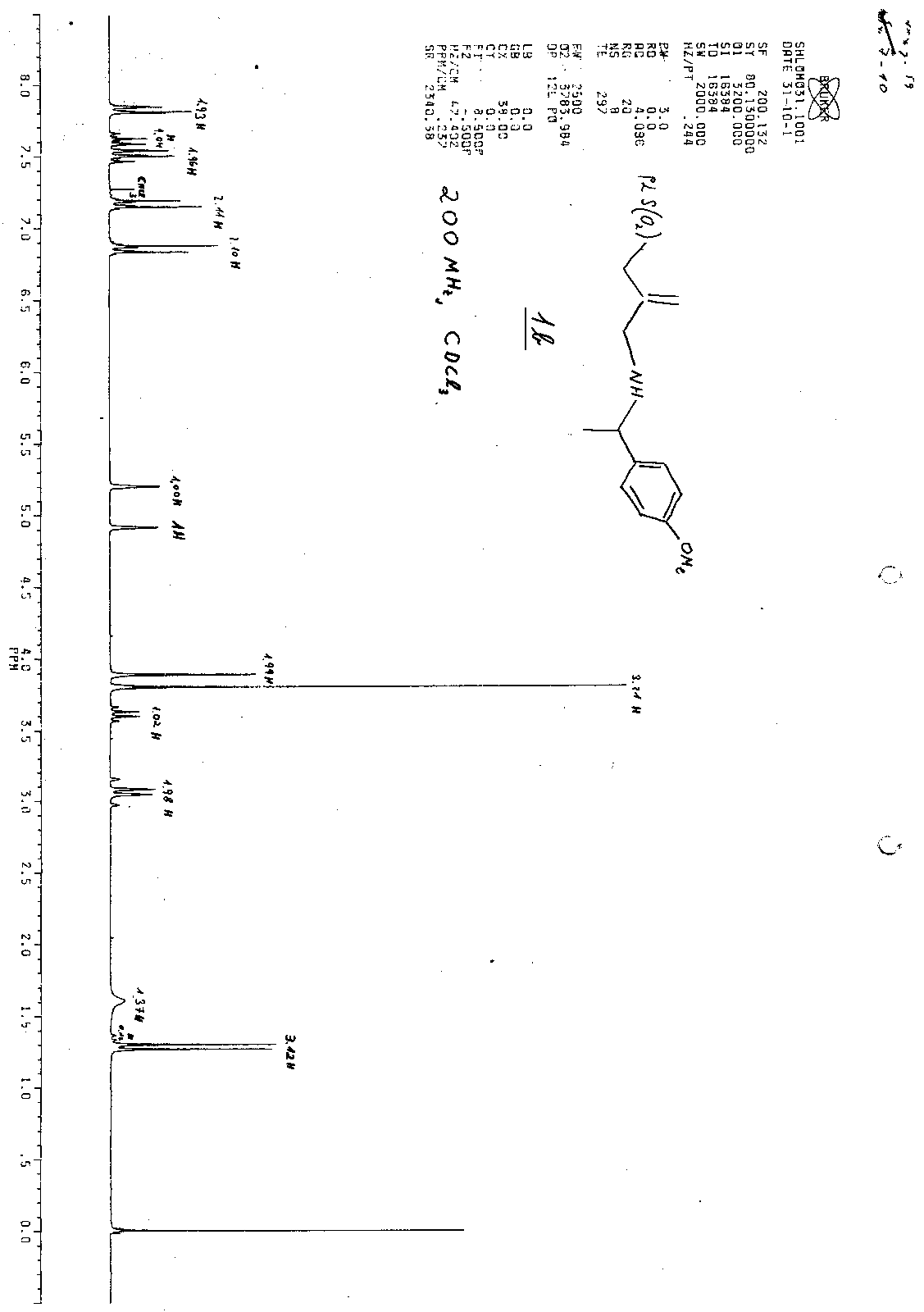

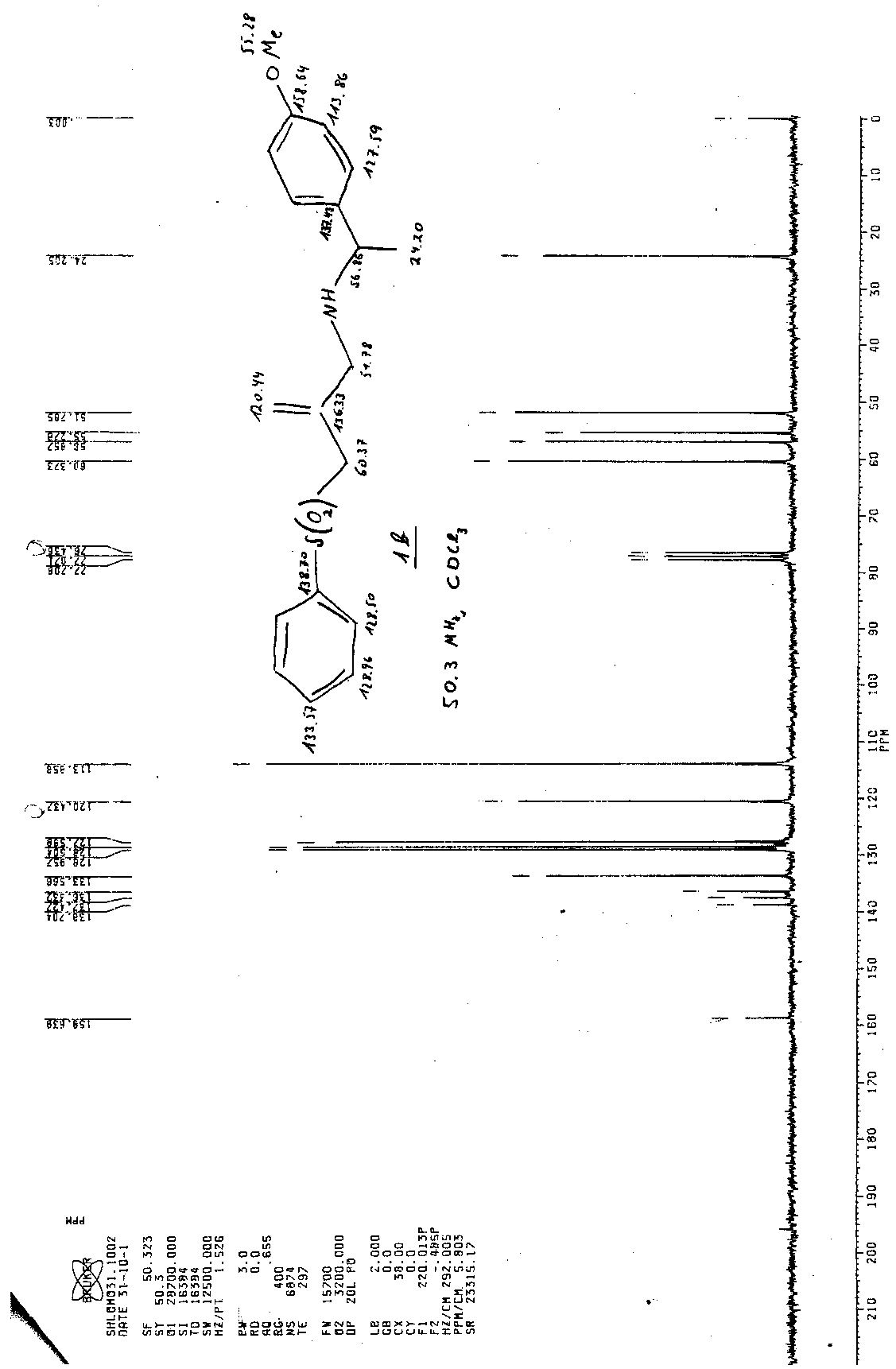

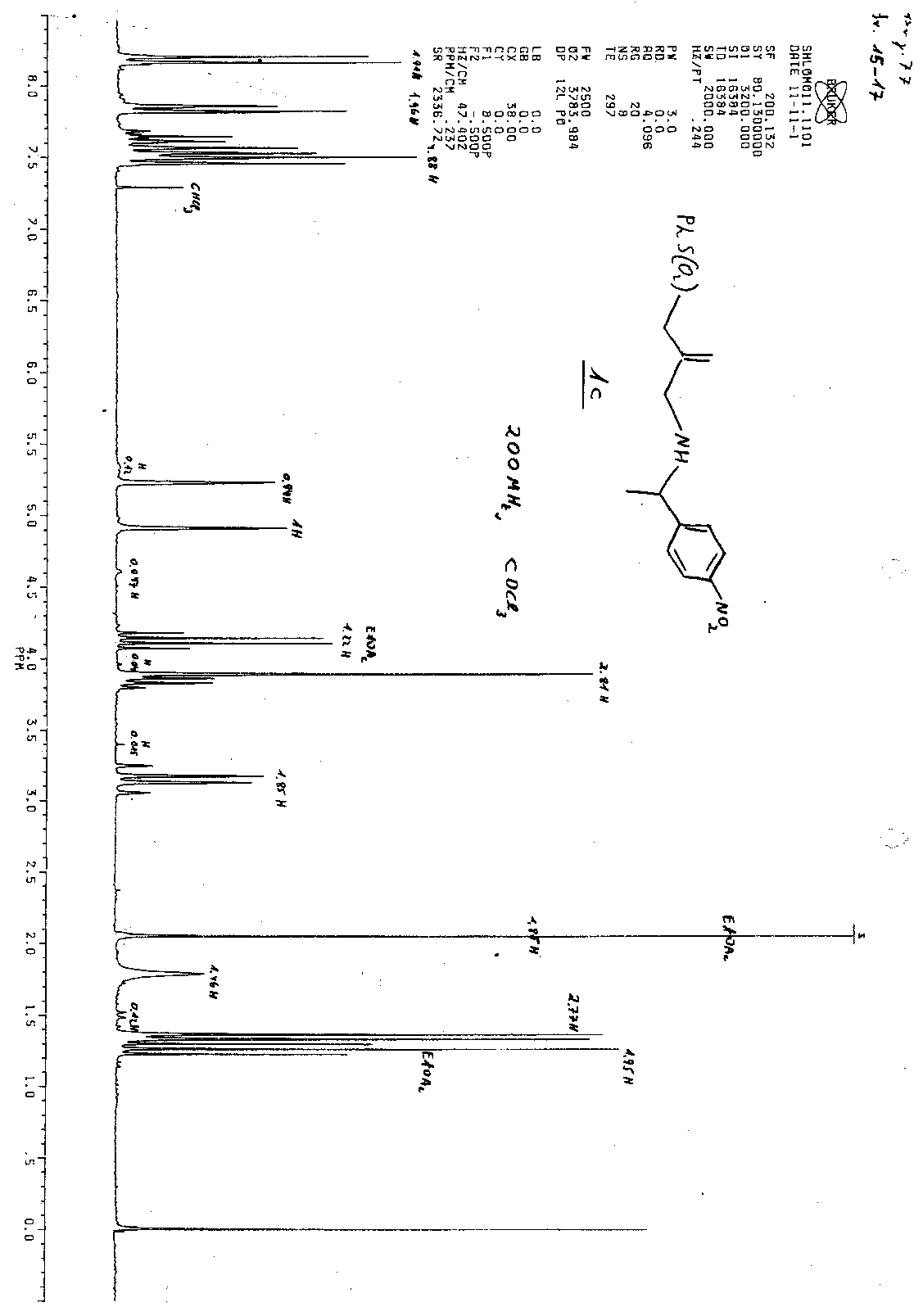

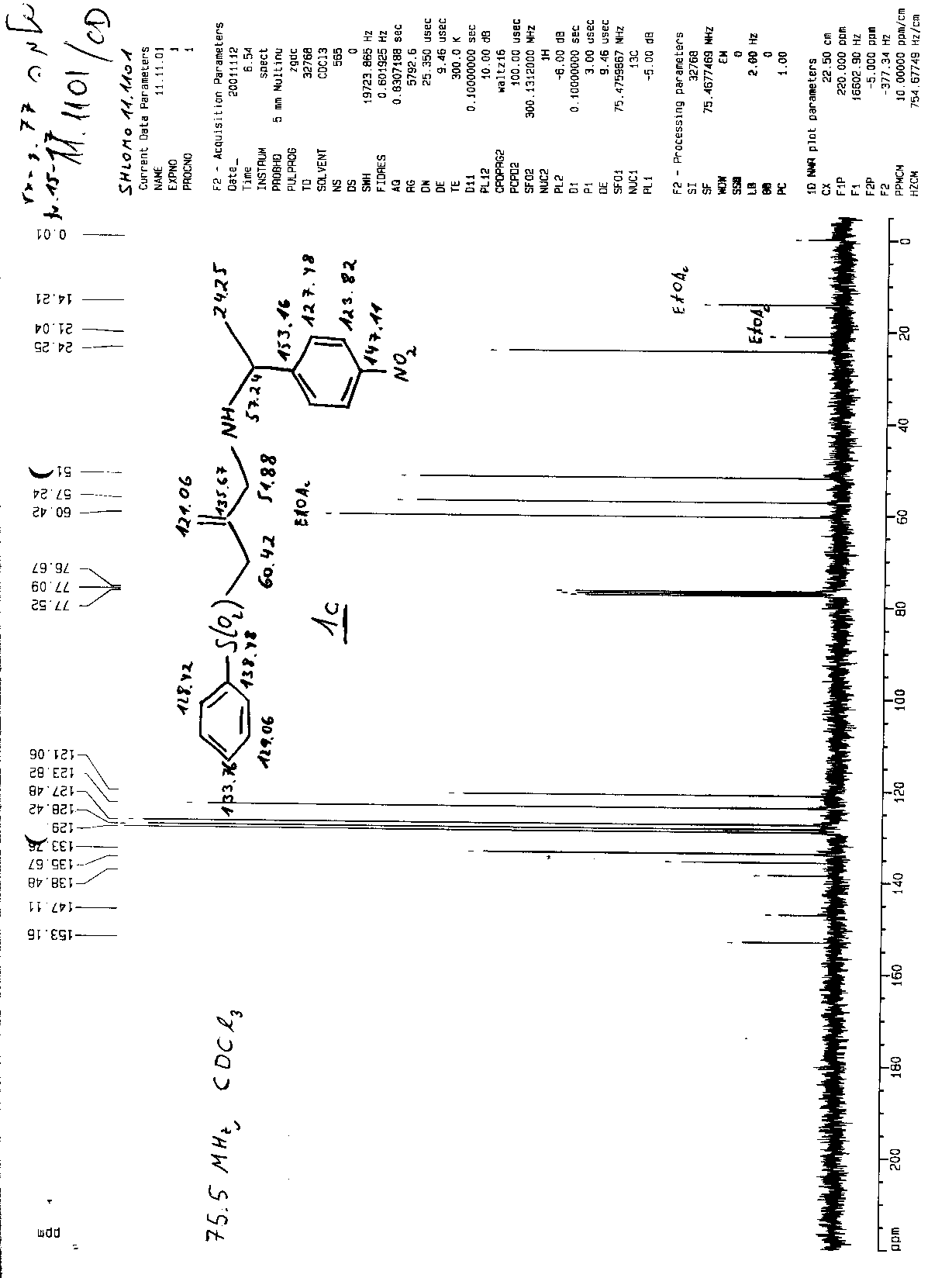

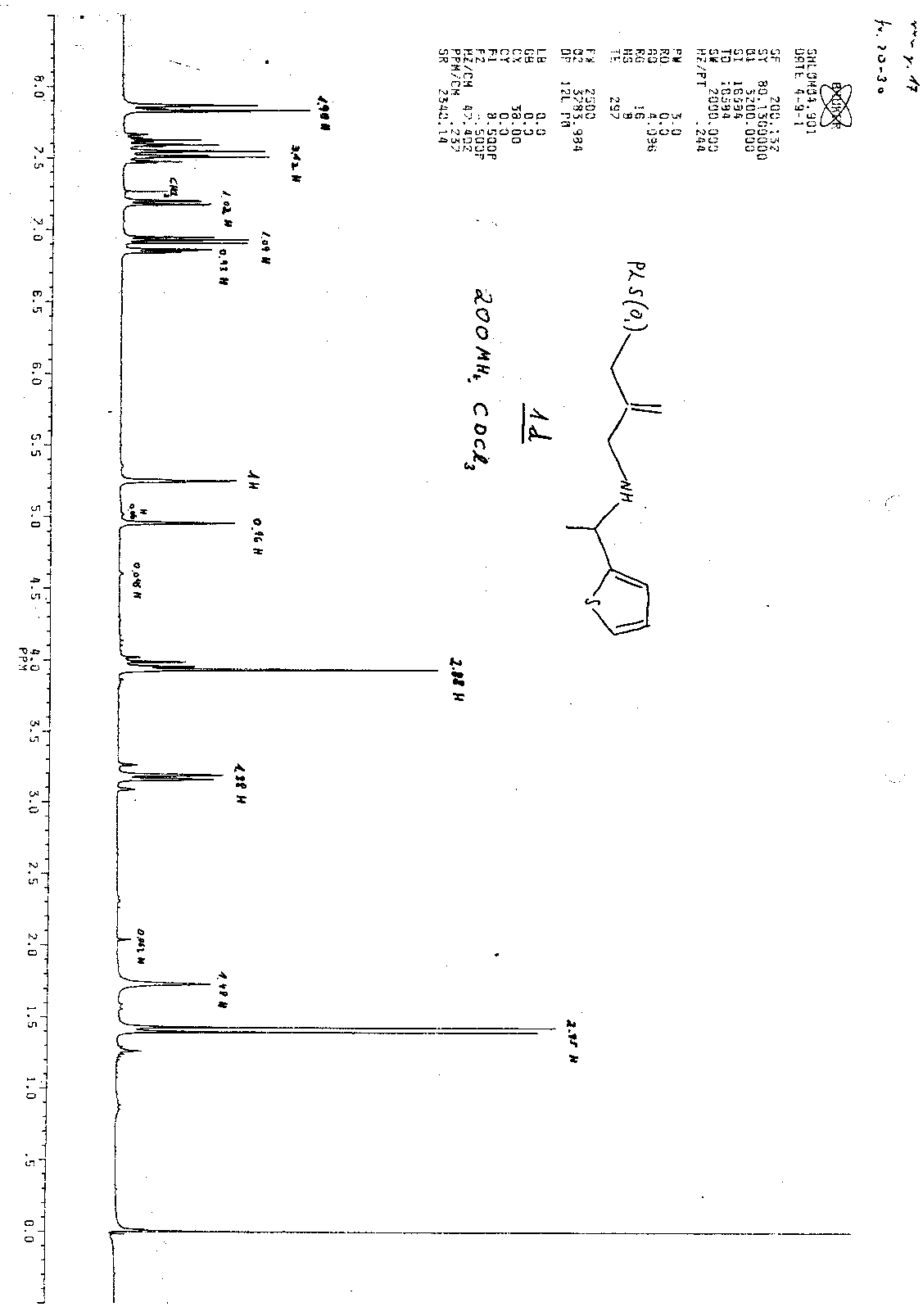

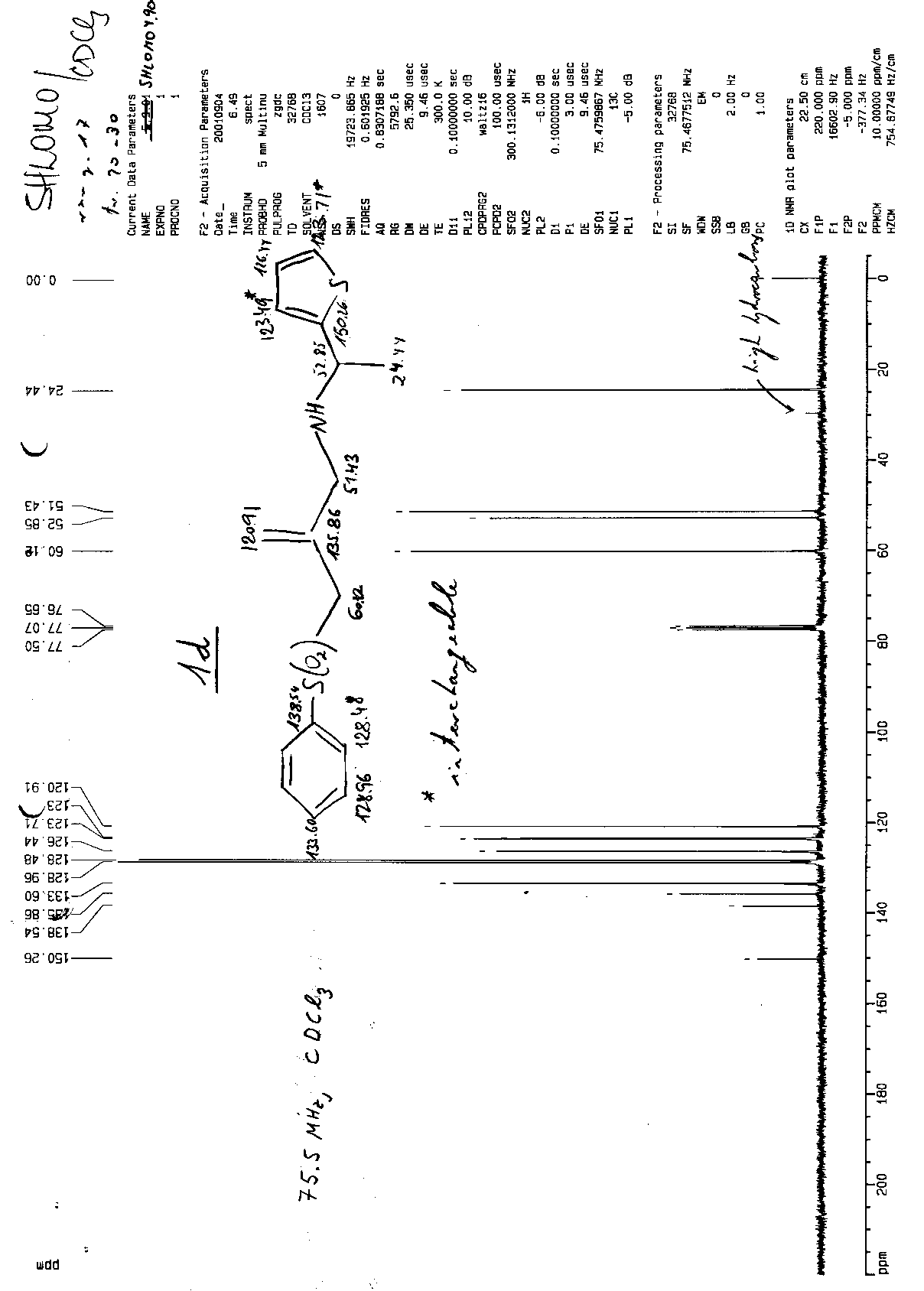

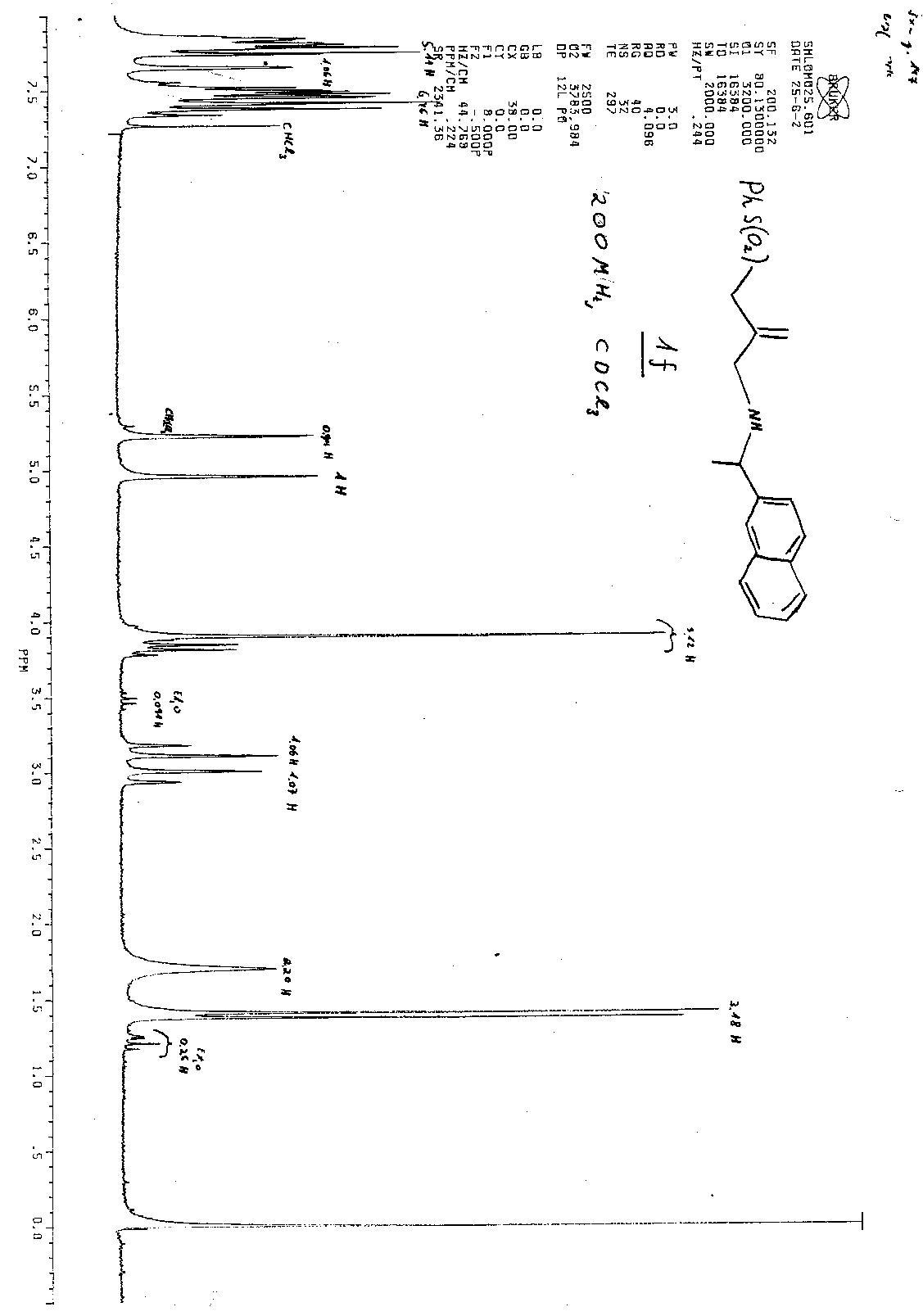

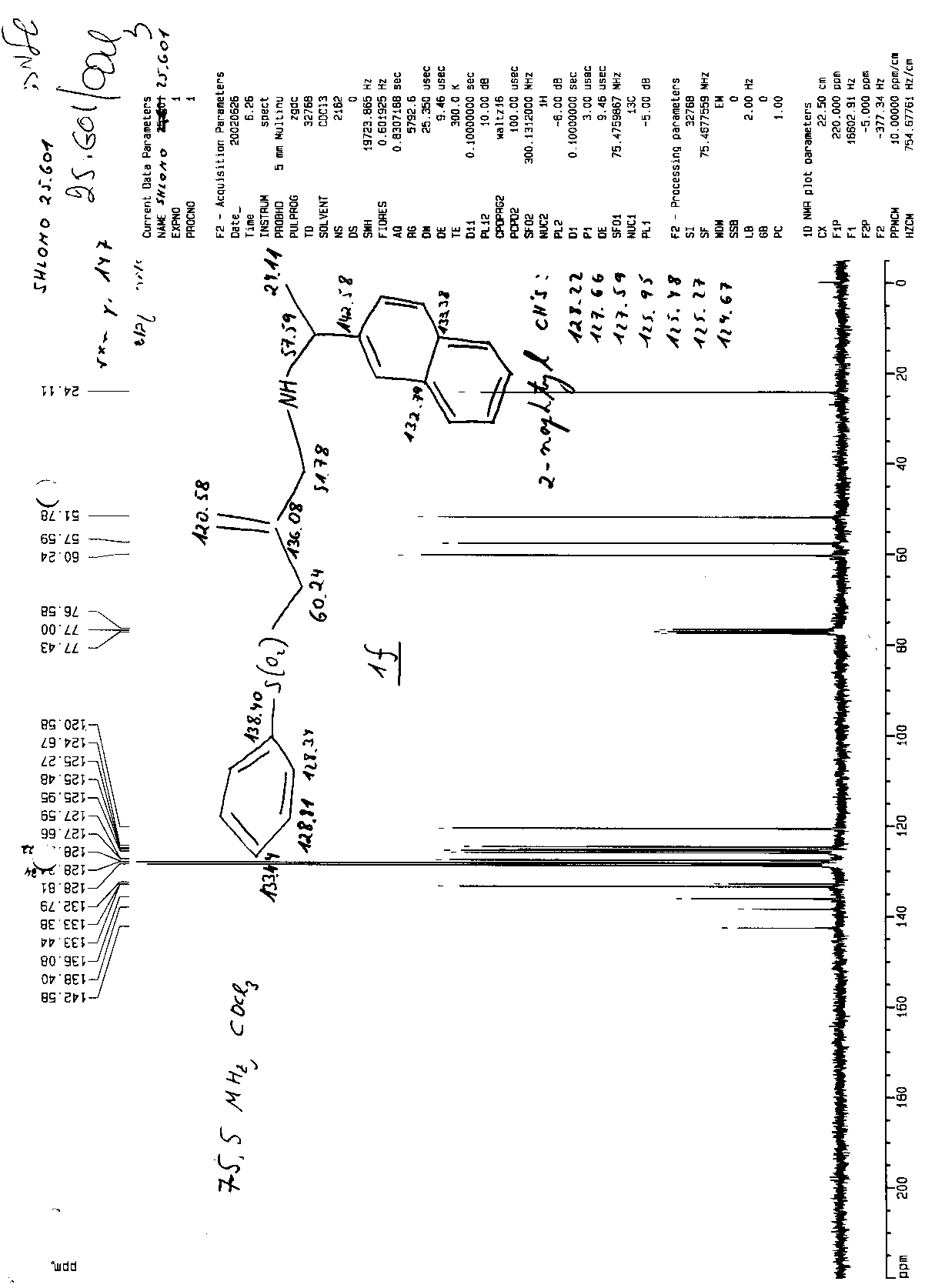

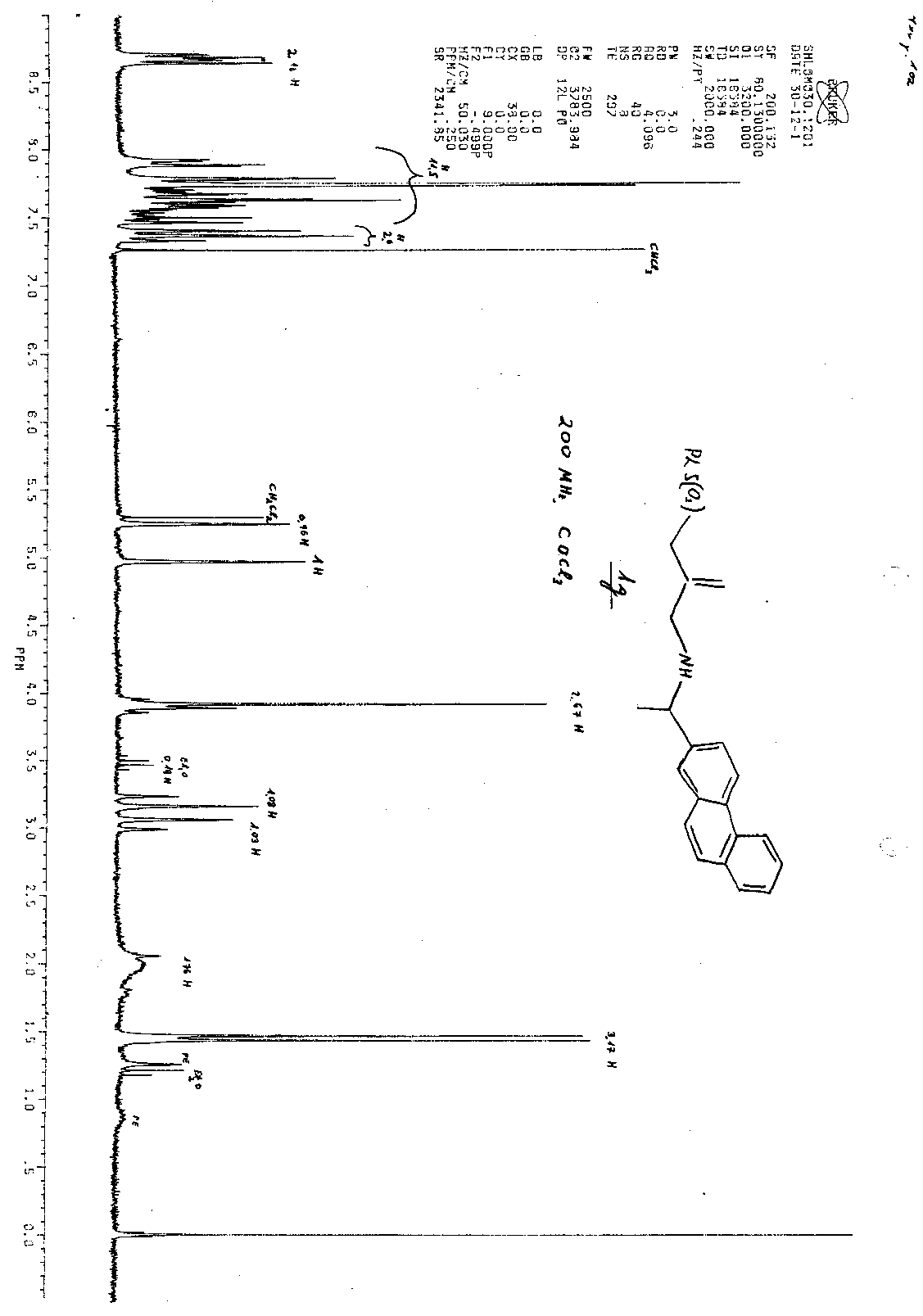

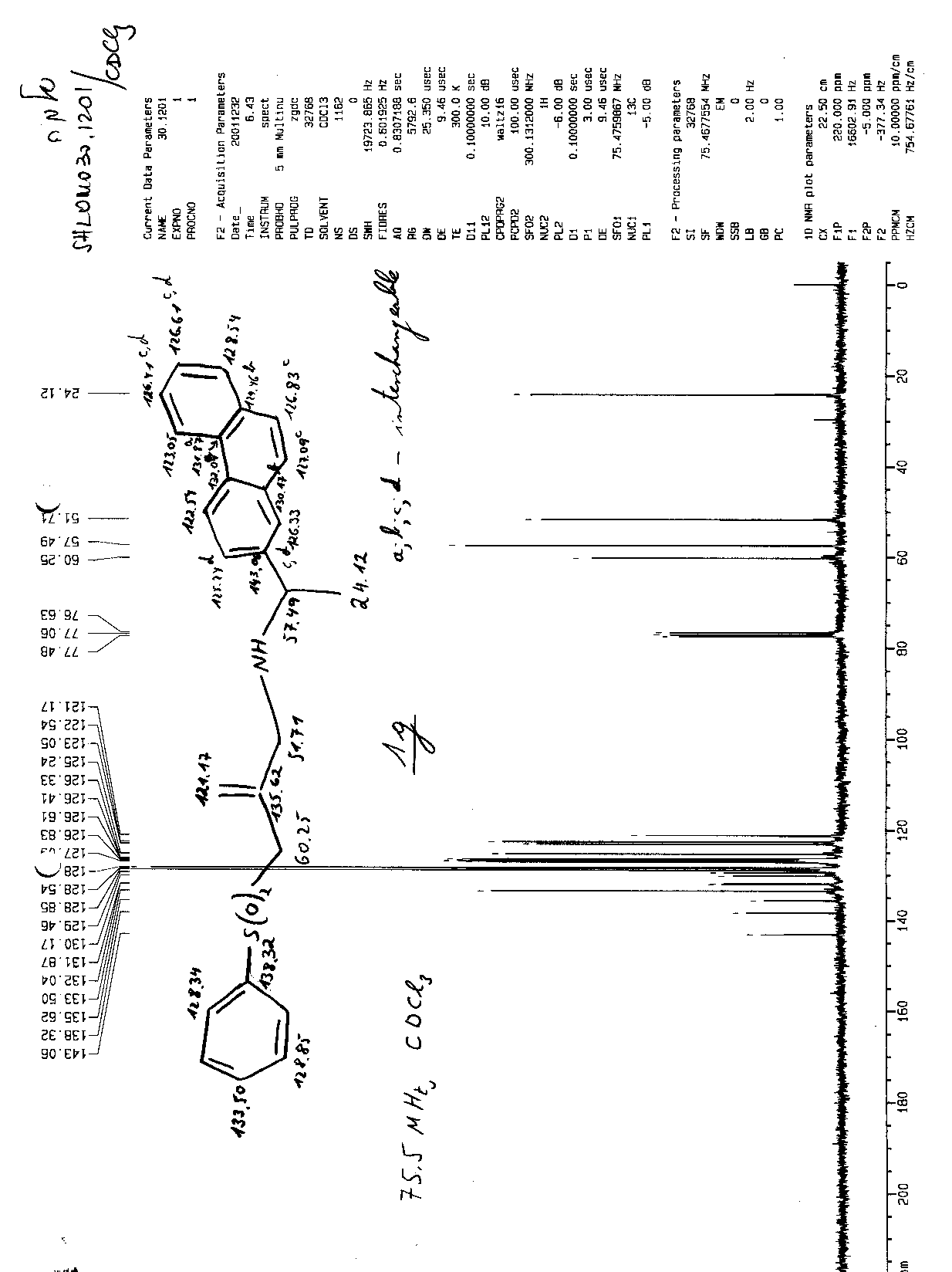

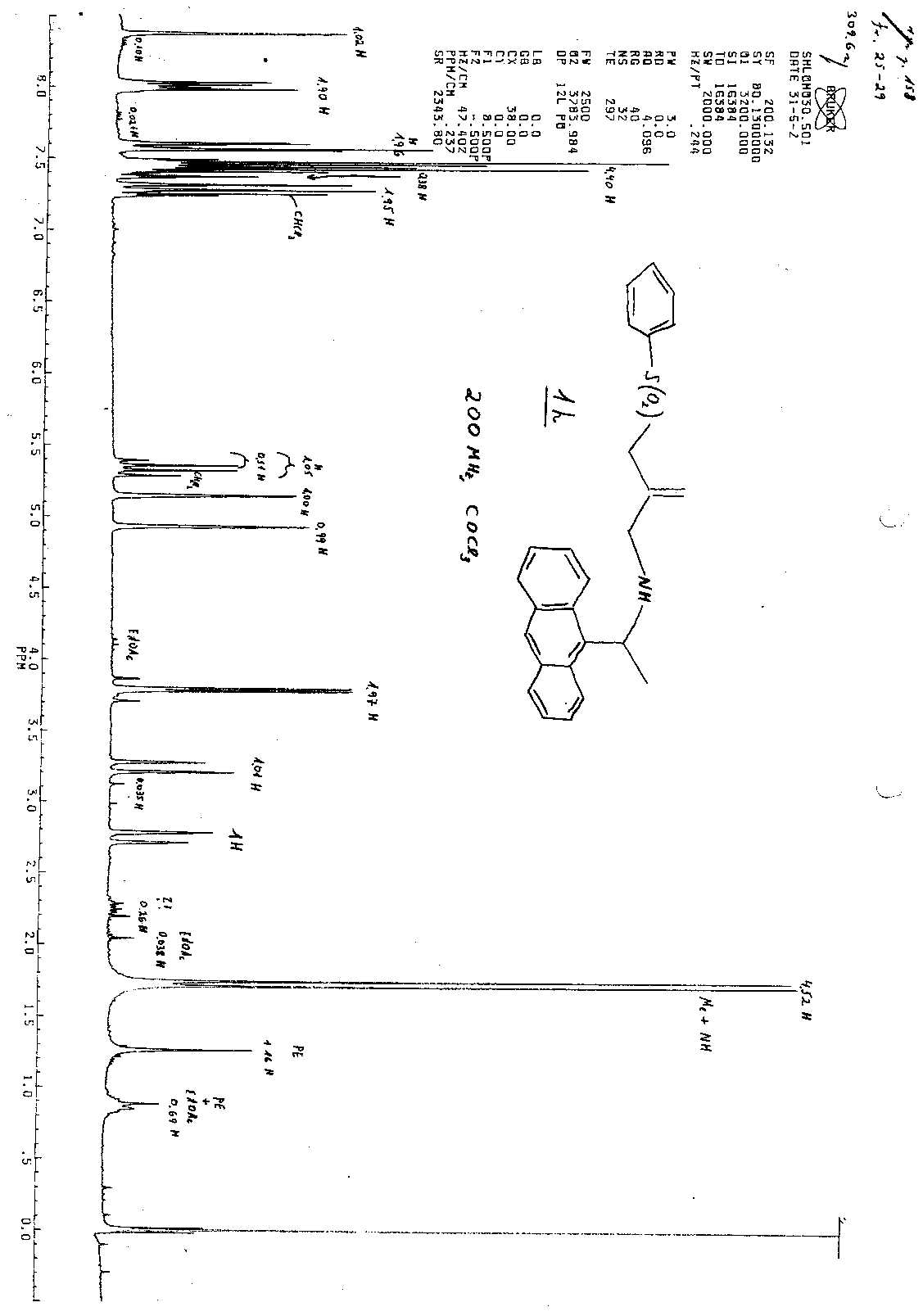

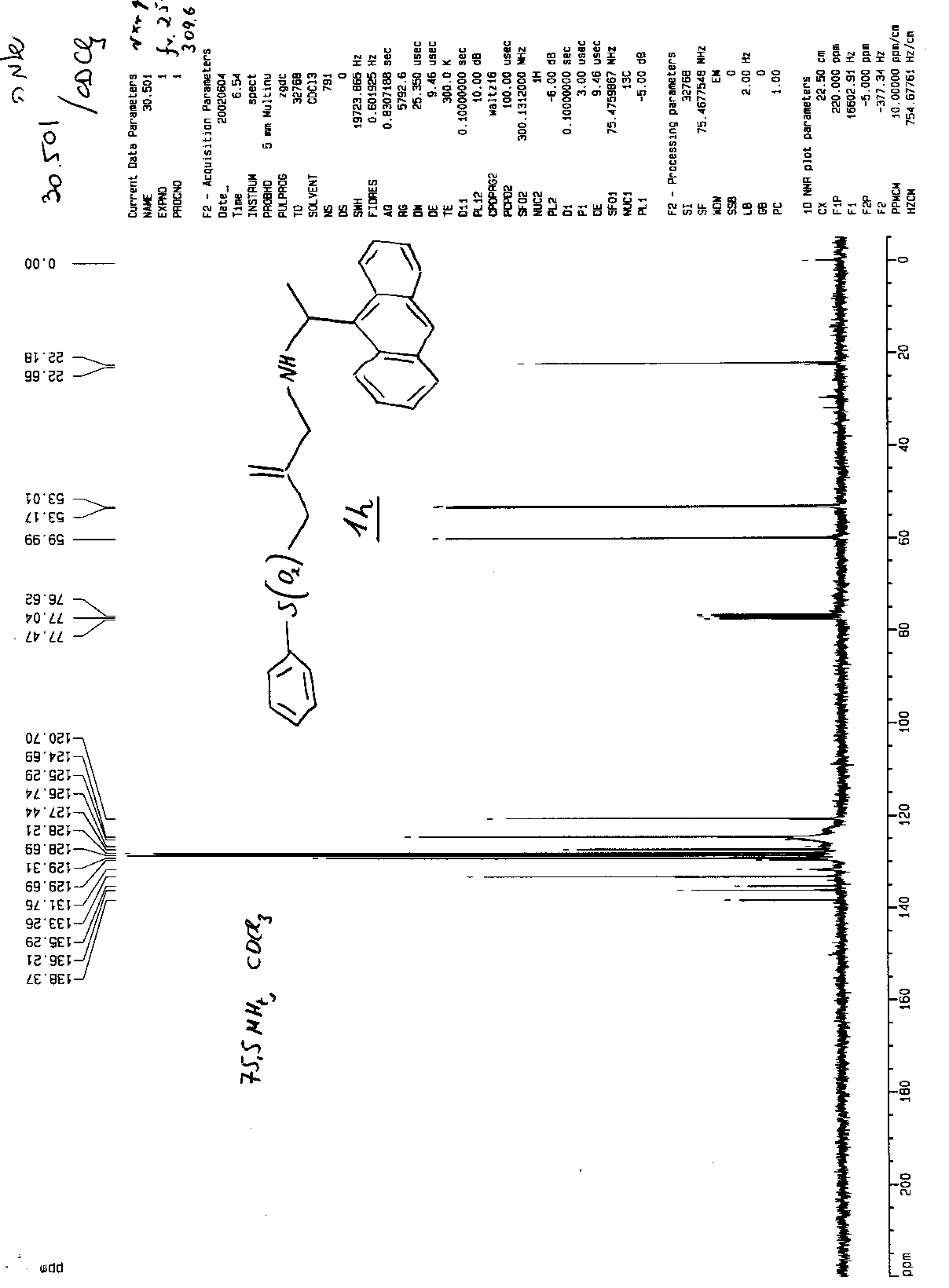

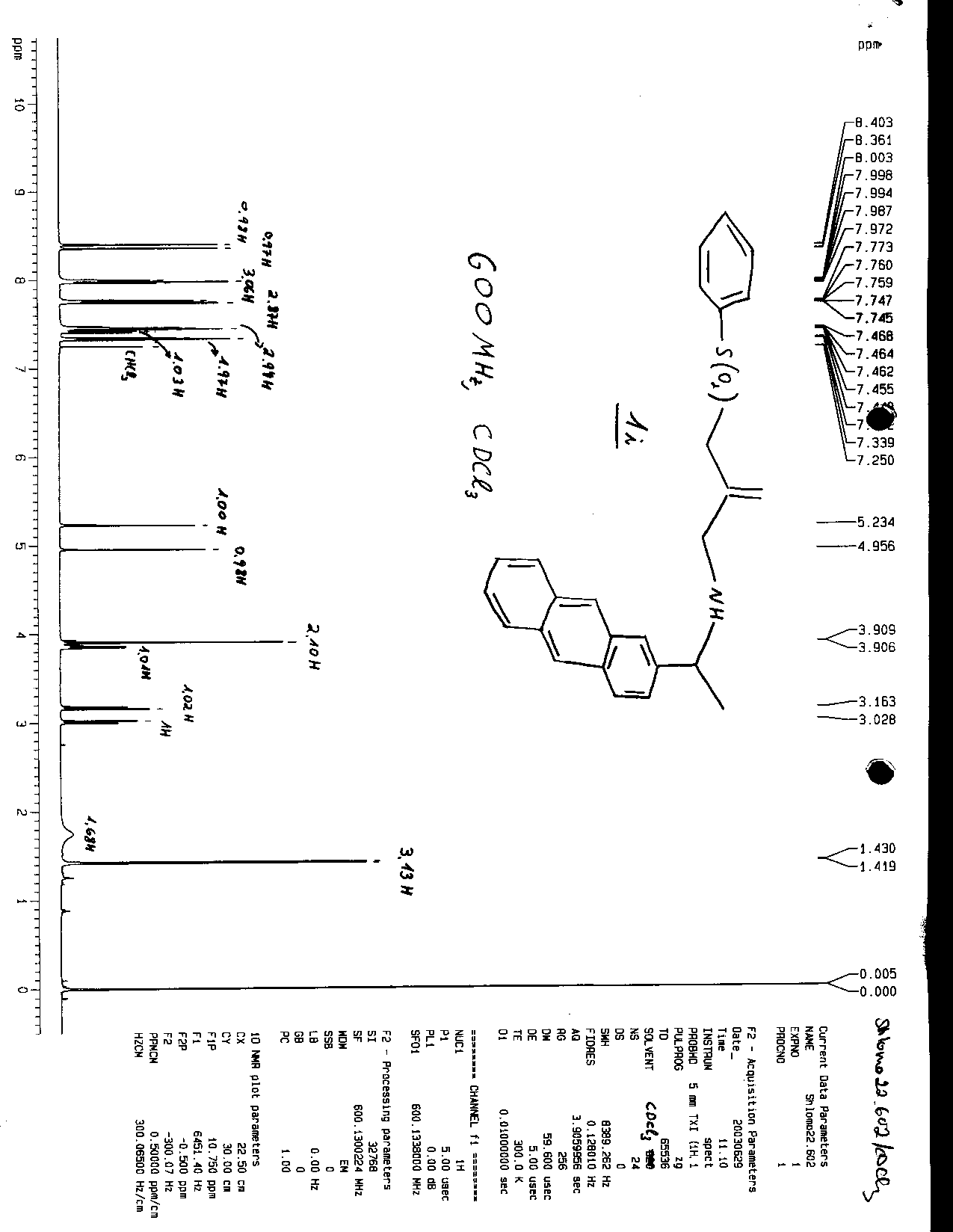

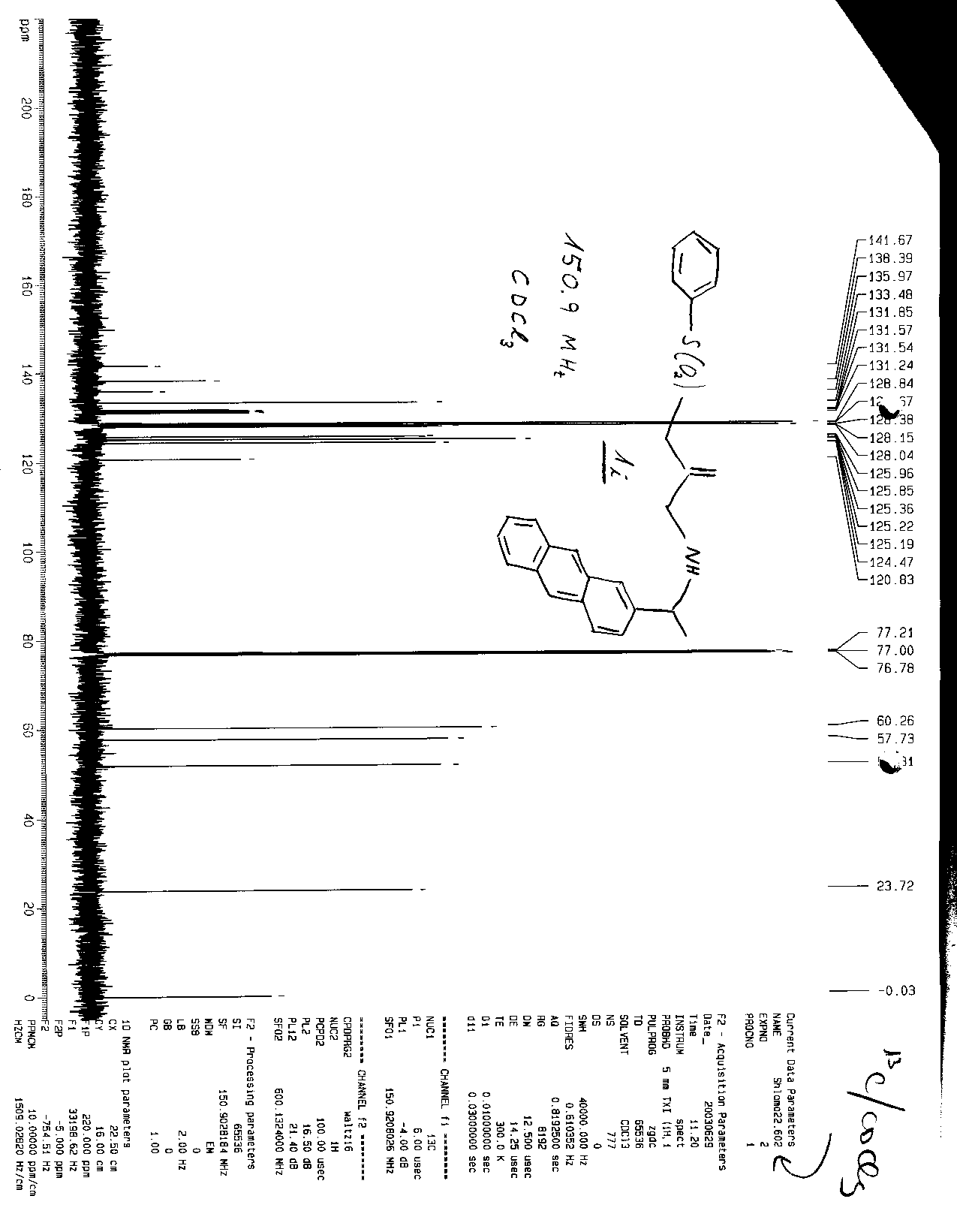

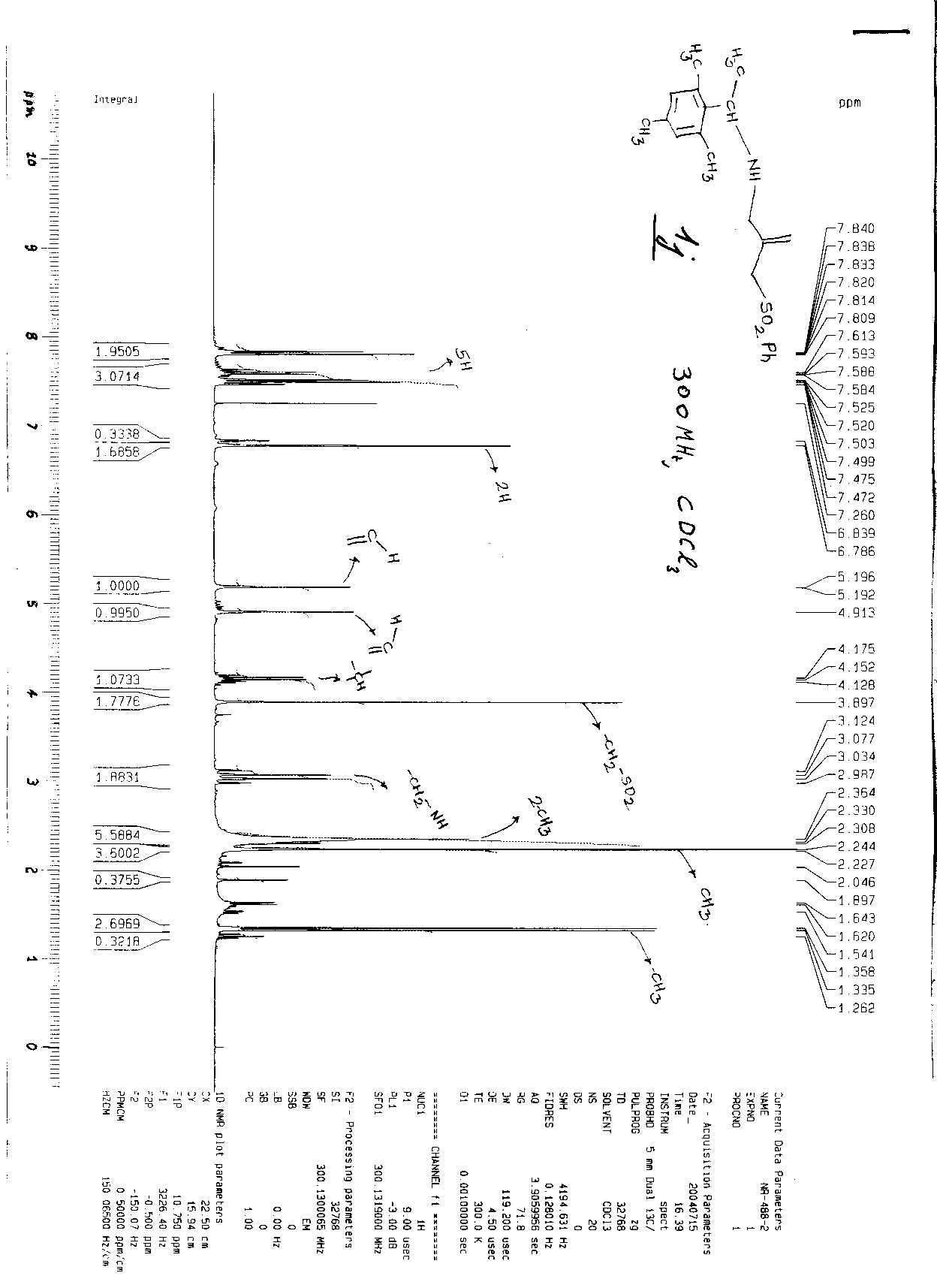

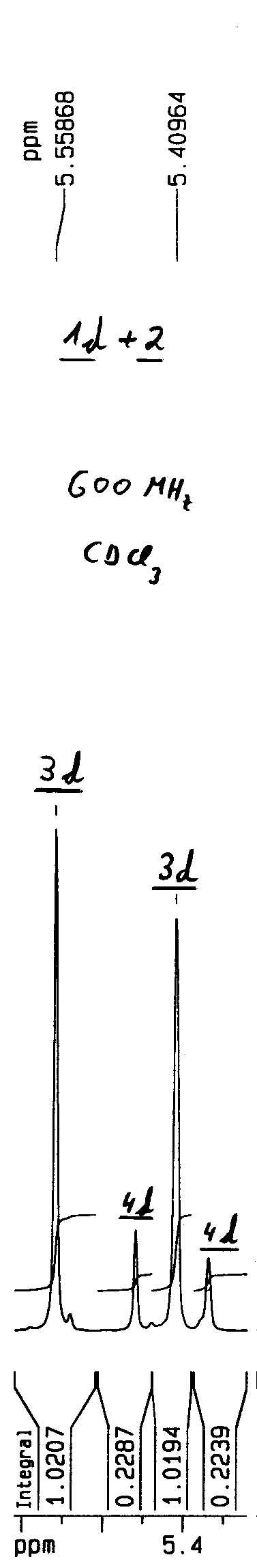

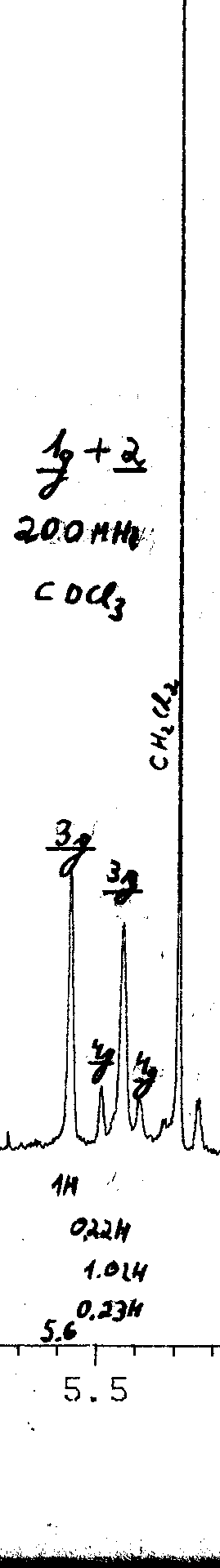


(after chromatography

of the crude product mixture)


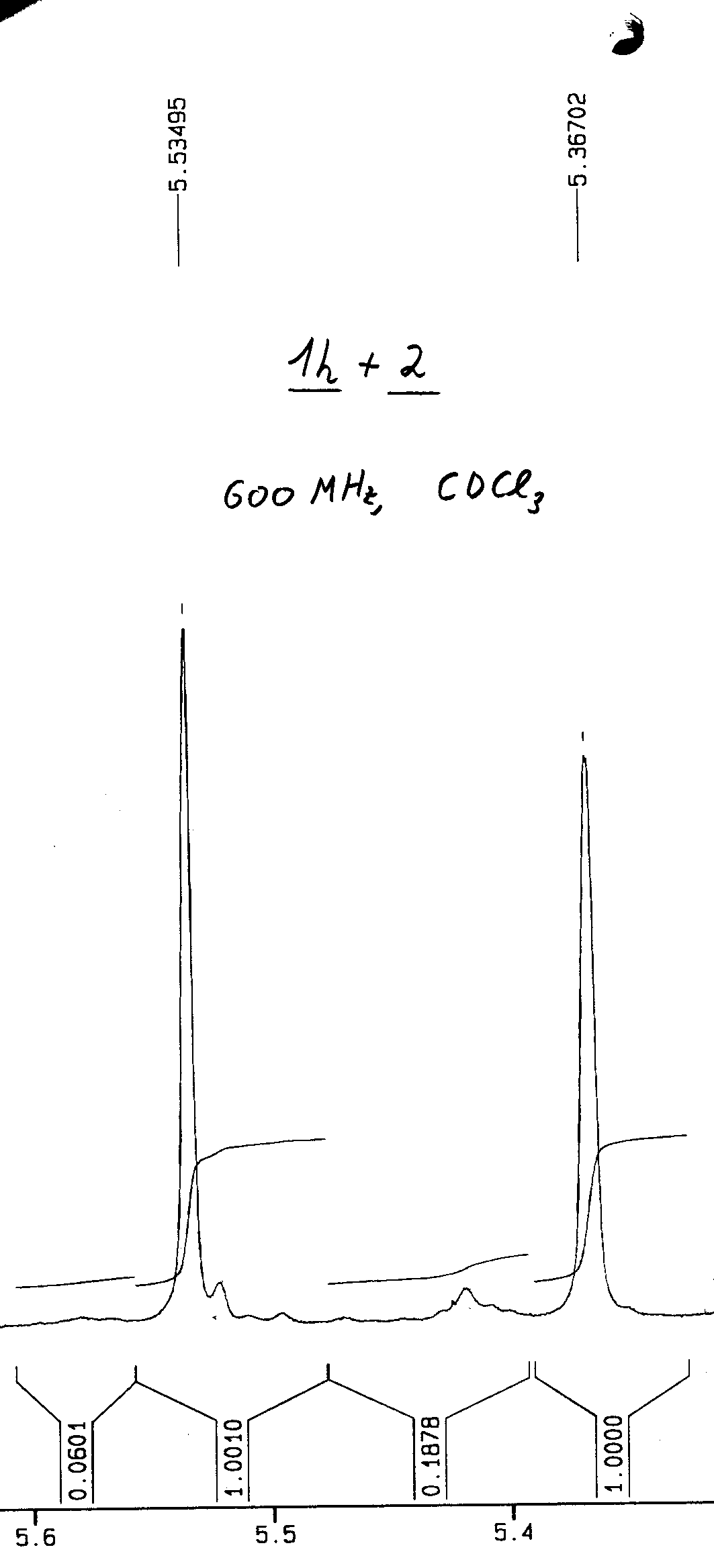

Supplement: File 1 — Experimental procedures, characterization data and NMR spectra. [file Beilstein_J_Org_Chem-04-32-s001.doc]
